# Guidelines for the diagnosis and treatment of knee osteoarthritis with integrative medicine based on traditional Chinese medicine

**DOI:** 10.3389/fmed.2023.1260943

**Published:** 2023-10-17

**Authors:** Lingfeng Zeng, Guanghui Zhou, Weiyi Yang, Jun Liu

**Affiliations:** ^1^State Key Laboratory of Traditional Chinese Medicine Syndrome/The Second Clinical College of Guangzhou University of Chinese Medicine, Guangzhou, China; ^2^The Second Affiliated Hospital of Guangzhou University of Chinese Medicine, Guangzhou, China; ^3^Bone and Joint Research Team of Degeneration and Injury, Guangdong Provincial Academy of Chinese Medical Sciences, Guangzhou, China; ^4^Guangdong Second Traditional Chinese Medicine Hospital (Guangdong Province Enginering Technology Research Institute of Traditional Chinese Medicine), Guangzhou, China

**Keywords:** degenerative arthritis, musculoskeletal diseases, bone aging, knee osteoarthritis, plant-based natural products, herbal medicine, evidence-based, traditional Chinese medicine

## Abstract

Knee osteoarthritis (KOA) is a common geriatric disease in middle-aged and elderly people. Its main pathological characteristics are articular cartilage degeneration, changes in subchondral bone reactivity, osteophyte formation at joint edges, synovial disease, ligament relaxation or contracture, and joint capsular contracture. The prevalence rate of symptomatic KOA in middle-aged and elderly people in China is 8.1%, and this is increasing. The main clinical manifestations of this disease are pain and limited activity of the knee joint, which seriously affect the quality of life of patients and may cause disability, posing a huge burden on society and the economy. Although the pathogenesis of KOA is not clear, the treatment of KOA is diverse, and Chinese medicine, which mainly relies on plant-based natural products, has a relatively stable and reliable curative effect. This guideline aims to emphasize the evidence-based staging and stepped treatment of KOA and the therapeutic effect of integrative medicine based on traditional Chinese medicine on KOA. We make recommendations that include the adoption of manual therapy, acupuncture, external application of herbs, herbal plasters, exercise therapy, and other integrative medicine based on traditional Chinese medicine. Users of the above guidelines are most likely to include clinicians and health managers in healthcare settings.

## Highlights

- This document discusses the diagnosis, differentiation, treatment, and health management of knee osteoarthritis.
- This document is applicable to the diagnosis and treatment of knee osteoarthritis.
- This document is suitable for use by clinicians in orthopedics, traditional Chinese medicine, acupuncture, manual therapy, rheumatology and immunology, rehabilitation, and other related departments.

## Introduction

Knee osteoarthritis (KOA) is classified into the “bone impediment” and “bi syndrome” categories in traditional Chinese medicine (TCM) (1–3). It is a chronic joint disease characterized by articular cartilage degeneration, subchondral bone disease, and synovial inflammation. In the early stage, the main symptoms are knee pain and tenderness, which are obvious when moving down stairs or performing strenuous activity, and in the late stage, joint movement limitation, muscular atrophy, and knee inversion deformity can occur. The prevalence of KOA is 24.7% in men and 54.6% in women aged over 40 years, and the final disability rate of the disease is 53% (2, 3). With the aging population, the incidence of this disease is on the rise. Recent research suggests that nearly half of people over the age of 60 and more than 80% of people over the age of 75 suffer from KOA (2, 3). Age, obesity, inflammation, trauma, and genetic factors may be related to the onset of KOA, which is characterized by primary or secondary degeneration of knee cartilage and bone hyperplasia.

Although there is currently no treatment that can reverse the progression of osteoarthritis, both pharmacological and non-pharmacological interventions that can alleviate these symptoms are being used (1–5). Clinicians practicing “integrative medicine based on traditional Chinese medicine” pay attention to the needs that are not being met by current interventions. We call these “unmet medical needs,” and in fact, it is considered that the purpose of all traditional medicine in modern society is to meet these needs. TCM-based integrative medicine has an advantage in that it can solve some problems in patients with KOA.

Regarding the diagnosis and treatment of KOA, the relevant guidelines issued in China include the *Clinical Diagnosis and Treatment Guide* of the Chinese Medical Association, the *Diagnosis and Treatment Guide of Common Diseases of Orthopedics and Traumatology* of the Traditional Chinese Medicine Association, the *Guidelines for Integrative Diagnosis and Treatment of Knee Osteoarthritis* of the Chinese Integrative Medicine Association, and the *Diagnosis and Treatment Guide of Osteoarthritis* of the Chinese Rheumatology Association. This guide highlights the staged treatment of KOA more than previous versions and previous guidelines. As clinical and basic research advances, recommendations need to be updated. Therefore, the development of clinical practice guidelines for the integration of TCM and Western medicine for KOA based on evidence-based medicine is of great significance, which helps to implement the principles of evidence-based medicine in clinical practice, standardize the clinical diagnosis and treatment techniques of integrative medicine based on TCM, promote the quality of medical services, help clinicians and patients choose the best treatment plan, and achieve better curative effects.

We developed this guideline using a systematic methodology to summarize and evaluate the effects of integrative TCM-based KOA treatment. We hope this guideline provides guidance to prevent and treat KOA in clinical practice. Due to the limitation on the length of this paper, the relevant research methods and procedures are provided in the Supplementary material.

This diagnosis and treatment guide is compiled by referring to the latest international and Chinese guidelines, bringing together the diagnosis and treatment experience and research results of experts in TCM and Western medicine, and it strives to explain the principles of TCM and Western medicine treatment in different periods of the disease in a concise manner. To assist clinicians and TCM doctors, as well as rehabilitative and nursing staff, to better apply the diagnosis and treatment guidelines of integrative medicine based on TCM to the treatment of patients with KOA, its scientific background, practical value, and compliance need to be continuously verified in clinical practice and updated and improved according to feedback from clinical practice.

## Diagnosis

### Medical history

Patients have a history of strain such as excessive weight bearing of the knee joint, congenital malformations around the knee joint (varus, valgus, etc.), or a history of trauma. Patients are mostly middle-aged and elderly.

### Signs and symptoms

1. Pain and tenderness: The incidence of pain and tenderness is 36.8–60.7% (2). The following types of pain occur: ① starting pain: after sitting for a long time or when just getting out of bed, it hurts to start walking, and this pain is slightly relieved after activity; ② activity pain: after walking for a period of time, the pain intensifies; ③ weight-bearing pain: pain occurs when the knee joint is in a weight-bearing state, such as when going up and down stairs; and ④ resting pain: the knee joint in the resting state is painful, especially at night. In addition to pain, local tenderness may appear in the knee joint, which is obvious when the joint is swollen (2, 3).
2. Limited activity: There is joint stiffness and tightness in the morning, which often lasts a few minutes to 10 min, and rarely more than 30 min. Joint lock can gradually appear, late joint activity is obviously limited, and this can eventually disable the patient (3).
3. Joint deformities: In the late stage of the disease, obvious internal, eversion, or rotation deformities can be seen.
4. Bone rub feeling: Joint flexion and extension and bone friction sounds can be heard.
5. Muscle atrophy: Atrophy of the extensor muscle group around the knee joint can occur (3), with extensor atrophy being the most significant (4, 5).

### Laboratory examination

In the acute phase, C-reactive protein (CRP) and the erythrocyte sedimentation rate (ESR) may be slightly elevated, but other blood values, immune complexes, and serum complement factor levels may be normal (1, 2).

Patients with synovitis may have joint effusion. The joint fluid is transparent and light yellow, and its viscosity is normal or slightly reduced. Joint fluid routine examination can show mild leukocytosis, mainly monocytes. Synovial fluid analysis helps rule out other joint diseases (2, 3).

### Imaging examination

Imaging can help to not only diagnose osteoarthritis but also to assess the severity of joint injury, to evaluate disease progression and response to treatment, and to aid in early detection of disease or related complications. This guide describes the grading criteria for X-ray and magnetic resonance imaging (MRI) commonly used in the diagnosis of KOA.

#### X-ray

Full-length weight-bearing radiographs of the lower limbs of the knee joint, anterior-lateral radiographs of the knee joint, and axial radiographs of the patella are conventionally the preferred images (6). In the early stage, radiographs are usually normal, and in the middle and late stages, asymmetric narrowing of the joint space, subchondral osteosclerosis and/or cystic changes, joint edge hyperplasia and osteophyte formation, and free bodies can be seen. For imaging classification, we refer to the Kellgren–Lawrence image classification method (7), as shown in Table 1.

**Table 1:** Kellgren–Lawrence knee osteoarthritis grading method.

Grade	Joint change
Grade 0	No changes
Grade I	Slight joint space narrowing and possible osteophytic lipping
Grade II	Presence of osteophytes and possible narrowing of the joint space
Grade III	Presence of multiple osteophytes, definite narrowing of the joint space, some sclerosis, and possible deformity of the bone ends
Grade IV	Presence of large osteophytes, marked narrowing of the joint space, severe sclerosis, and definite deformity of bone ends

#### MRI

MRI helps to detect and assess the extent of lesions in the joint-related tissues, such as cartilage injury, joint synovial effusion, subchondral bone marrow edema, synovitis, and meniscus or ligament injury. It can also be used to exclude tumors and ischemic osteonecrosis. Generally, Recht grading is the standard for MRI (8), as shown in Table 2.

**Table 2:** Recht grading of MRI for knee osteoarthritis.

Grade	Imaging manifestations
Grade 0	No change (normal)
Grade I	Abnormal signals within the cartilage, but the cartilage surface is smooth
Grade II	Mild irregularities on the surface of the cartilage and/or focal defects of less than 50% of the full thickness of the cartilage
Grade III	The surface of the cartilage is severely irregular and/or focal defects of more than 50% of the total thickness of the cartilage but less than the full thickness occur
Grade IV	Full-layer cartilage defect with exposed subchondral bone

### Diagnostic criteria

Diagnostic criteria are mainly based on the patient’s symptoms, signs, and imaging results. Here, the diagnostic criteria revised by the Chinese Association of Integrative Medicine Based on Traditional Chinese Medicine in 2018 are adopted (9), as shown in Table 3.

**Table 3:** Classification criteria of knee osteoarthritis.

Sequence number	Symptoms or signs
1	Recurring knee pain in the past month
2	Age ≥ 50 years
3	Morning stiffness time ≤ 30 min
4	Bone friction while moving the knee
5	X-ray (standing or weight-bearing position) shows narrowing of the joint space, subchondral bone sclerosis and/or cystic degeneration, and osteophyte formation at the joint margin
6	MRI shows cartilage injury, osteophyte formation, subchondral bone marrow edema and/or cystic change, degenerative meniscal tear, and partial or full cartilage loss

Knee osteoarthritis can be diagnosed if diagnostic criteria 1 + 2 + 3 + 4 or 1 + 5 or 1 + 6 are met.

### Differential diagnosis of KOA

Knee osteoarthritis should not be confused with the following diseases (10).

#### Rheumatoid arthritis

Most cases of rheumatoid arthritis are symmetrical microarthritis, mainly involving proximal interphalangeal joints, metacarpophalangeal joints, and wrist joints, with obvious morning stiffness. Subcutaneous nodules may occur, and they may be rheumatoid factor-positive. X-ray examination shows mainly joint erosive change. Other indications of rheumatoid arthritis include the presence of anti-cyclic peptide containing citrulline (anti-CCP) and lung involvement, for example interstitial lung disease. Moreover, RA can begin as monoarthritis in the elderly, often involving knee or other big joints (2, 3).

#### Gouty arthritis

In middle-aged and elderly people, mostly men, recurrent acute arthritis may occur, which most often involves the first metatarsophalangeal joint and tarsal joint, and sometimes also the knee, ankle, elbow, wrist, and hand joints. It presents with joint redness, swelling, heat, and severe pain. The blood uric acid level is elevated and urate crystals can be found in the synovial fluid. In chronic cases, kidney damage may occur, and gout stones may appear around the joints and in the auricle (3, 6).

#### Ankylosing spondylitis

Ankylosing spondylitis mostly occurs in young men and mainly affects the sacroiliac joints and spine. Knee, ankle, and hip joints are often involved, morning stiffness is obvious, patients often have inflammatory low back pain, radiological examination often reveals sacroiliarthritis, and patients are often positive for human leukocyte antigen (HLA)-B27 (6, 9).

#### Psoriatic arthritis

Psoriatic arthritis usually occurs in middle-aged people, and the onset is slow, mainly involving the limbs joints of the distal finger (toe), metacarpal and phalangeal joints, metatarsal joints, and knee and wrist joints. The joint lesions are often asymmetric, and the joints can be deformed. During the course of the disease, psoriasis may cause changes in the skin and nails of the fingers (toes) (9, 10).

## Chronic disease management

Knee osteoarthritis should be included in chronic disease management. Chronic disease management includes regular detection of common symptoms, continuous monitoring, evaluation, and comprehensive intervention for KOA and its risk factors, as well as lifestyle management and the evaluation of management effects. Chronic disease management emphasizes health education and non-drug interventions at all stages of disease and development to maximize the self-management ability of patients in daily life.

The *AAOS Clinical Practice Guideline Summary: Management of Osteoarthritis of the Knee (Nonarthroplasty), Third Edition* (11) suggests that health education and lifestyle changes play an important role in the treatment of KOA and that orthopedic surgeons and other skeletal and musculoskeletal health professionals should treat arthritis as a chronic disease.

## Clinical stage and Chinese classification of KOA

### Clinical stage

Traditional Chinese medicine concepts and imaging results should be combined in KOA staging and classification for chronic disease management and treatment (12, 13). Under the holistic view of TCM and general health concepts, KOA is divided into five stages based on the theory of TCM treatment without disease, the modern concept of knee preservation, and the requirements of patient subdivision in chronic disease management, combined with imaging evaluation, as shown in Table 4 (Highly recommended).

**Table 4:** Imaging stages and clinical manifestations of knee osteoarthritis.

Stage	Clinical manifestation	Imaging manifestations	Time division
Stage I (pre-stage)	Joints have mild discomfort; patients experience cold intolerance; going up the stairs, squatting, and standing up is difficult; joints may have friction; and joint activity may make noise. Acute synovitis may occur in very few patients after intense exercise, but the diagnostic criteria do not include osteoarthritis or developmental internal and external joint inversion deformity.	Cartilage wear grade 0. The Kellgren–Lawrence imaging grade is 0 or I, and MRI findings are normal.	Pre-disease stage/erotogenic stage
Stage II (early stage)	In the early stage, knee osteoarthritis (KOA) can be confirmed by diagnostic criteria. Non-drug therapy can be given, and acute episodes of pain after excessive exercise or exertion can generally be cured clinically.	Meniscus injury stage. Meniscus degeneration, tear, or exophysis and subchondral bone marrow edema can occur, and symptoms of single ventricular high pressure may be revealed by imaging. MRI shows abnormal signals within the cartilage, but the surface of the cartilage is smooth. The Kellgren–Lawrence imaging grade is I or II.	Stage of attack/remission
Stage III (middle stage)	The number of acute episodes of joint pain and swelling increases, which require painkiller control, and the symptoms are not easy to cure, which require multiple long-term therapies.	Period of partial cartilage wear. Partial cartilage wear is accompanied by subchondral bone edema and meniscus degeneration, tear, or exophysis, and imaging may reveal symptoms of single ventricular hypertension. MRI shows mild irregularities on the surface of the cartilage and/or focal defects of less than 50% of the full thickness of the cartilage. The Kellgren–Lawrence imaging grade is II or III.	Stage of attack/remission
Stage IV (late stage)	The developmental articular inversion angle is increased. The number of acute attacks of joint pain and swelling increases, and the symptoms cannot be completely relieved by medication.	Single compartment contact phase. MRI shows serious irregularities on the surface of the cartilage and/or focal defects of more than 50% of the full thickness of the cartilage, bone marrow edema, and even local exposed and necrotic subchondral bone. The Kellgren–Lawrence imaging grade is III or IV.	Stage of attack/remission
Stage V (surgery stage)	The effect of conservative treatment is poor, the joints are stiff, movement is obviously impaired, swelling pain often occurs, muscle atrophy occurs, and a walker or a crutch is often needed to walk.	Multiple ventricular degeneration stage. The Kellgren–Lawrence imaging grade is IV. MRI shows extensive full-layer cartilage defects, exposed subchondral bone, and even bone necrosis.	Stage of attack/remission

Stage of attack: Knee joint pain is moderate or severe and persistent severe pain may cause sleep difficulties; knee joints are swollen, their function is limited, and patients limp or are even unable to walk. Remission: Mild pain in the knee joint is aggravated by fatigue or weather changes; patients may suffer from acid swelling, fatigue, or limited knee joint activity.

#### Evidence description

The previous three-stage method was not detailed enough to facilitate the management of chronic diseases. The stages of our guideline are based on the theory of treatment without disease, setting up the early stage (phase 1) to control risk factors, while the rest of the stages are subdivided into four phases according to the development of the disease, in order to achieve the management of the whole life cycle of chronic KOA, delay the degeneration and aging of the joint (including the postoperative period) as much as possible, prolong the service life of the joint, and improve the quality of life. The main consensus for regarding KOA classification is the five-stage clinical classification method, which is mainly based on the theory of Chinese treatment of non-disease, the modern concept of knee protection, and the requirements of chronic disease management in patient population segmentation (Highly recommended).

### Chinese classification

The syndrome differentiation methodology based on the *Guiding Principles for Clinical Research of New Chinese Medicine*, the *Classification and codes of diseases and patterns of traditional Chinese medicine*, and the *Guidelines for the Diagnosis and Treatment of Common Diseases of Orthopadics and Traumatology in traditional Chinese medicine* is further improved by the previous literature review, and the following syndrome types are summarized. There may be different syndromes or mixed syndromes, which can be differentiated according to clinical practice (14–18).

#### Dampness-cold obstruction syndrome

Traditional Chinese medicine pathogenesis: Cold and damp qi invade joints.
Main symptoms: The joint pain feels heavy; cold aggravates the feeling, and warmth reduces it.
Secondary symptoms: A heavy pain feeling in the waist, a pale tongue, greasy white fur, and a gentle pulse may occur.

#### Dampness-heat obstruction syndrome

Traditional Chinese medicine pathogenesis: Heat and damp qi invade joints.
Main symptoms: Joints are inflamed (redness of skin, swelling, heat, pain), flexion and extension are difficult, and walking is difficult.
Secondary symptoms: These include fever, thirst but no desire to drink, and being upset; the tongue is red, the fur is yellow and greasy, and the pulse is moist or slippery.

#### Qi-blood stagnation syndrome

Traditional Chinese medicine pathogenesis: Poor circulation of qi and blood.
Main symptoms: Joint pain and tingling may occur; after rest, the pain is worse.
Secondary symptoms: These include dark complexion, a dark purple tongue, and ecchymosis; the pulse may be heavy and uncomfortable.

#### Deficiency of liver and kidney

Traditional Chinese medicine pathogenesis: The function of the Zangfu is degraded, and the body is unable to nourish the bones and muscles.
Main symptom: Dull joint pain.
Secondary symptoms: These include waist and knee weakness and acid pain, which is even worse in case of labor. Moreover, the patient may have a red tongue with a small amount of coating; furthermore, their pulse may be thin and weak.

#### Qi-blood weakness syndrome

Traditional Chinese medicine pathogenesis: Deficiency of Qi and blood leads to a loss of nourishment of muscles and bones.
Main symptom: Joint pain and discomfort.
Secondary symptoms: These include less sleep and more dreams, uncontrollable sweating or night sweats, dizziness, palpitation, shortness of breath, a seedy look, a thin tongue, a thin white fur, and a thin and weak pulse.

## Treatment

### Therapeutic principle

Knee osteoarthritis treatment should follow the steps of TCM and Western medicine. KOA is a chronic degenerative joint disease, clinically divided into stage I (pre-stage), stage II (early stage), stage III (middle stage), stage IV (late stage), and stage V (surgery stage). The overall treatment method is a combination of non-drug and drug therapy, including surgical treatment if necessary, and treatment should be individualized. We should not only flexibly choose Chinese medicine, acupuncture, manual therapy, and other therapies according to the TCM system of syndrome differentiation and treatment but also carry out step treatment based on TCM and Western medicine and strictly grasp the indications. In step therapy, the treatment plan needs to be optimized continuously to maximize the curative effect. The comprehensive treatment effect of TCM is good throughout the entire course of treatment. Health education and exercise are important measures for treatment and consolidation of curative effects (12–14) (Highly recommended).

#### Description of the evidence

According to the clinical manifestations and imaging evaluation of KOA, KOA is divided into five stages for the staged TCM-based integrative medicine treatment. The clinical stages emphasize the concepts of “treatment without disease” and “prevention before disease” to actively prevent the occurrence of KOA by improving the patient’s lifestyle and avoiding risk factors. When KOA occurs, in order to prevent or delay the progression of KOA, the clinical manifestations and imaging data are integrated to provide individualized step therapy, making full use of the advantages of Chinese and Western medicine (19).

### Non-drug therapy

Non-drug therapy plays a very important role in the treatment of KOA throughout all clinical stages.

### Health education and self-management

Knee osteoarthritis patients should be educated about their health and helped to manage themselves.

Health education (20, 21): Health education can reduce pain and improve the psychosocial status of KOA patients. Doctors should guide patients to: ① recognize the disease and have a clear purpose of treatment (improve symptoms, delay the development of the disease); ② build confidence, eliminate the burden of thought, and relieve anxiety and fear of sports; ③ closely cooperate with doctors in diagnosis and treatment; and ④ adjust their lifestyle and perform reasonable exercise (Evidence level: Level II, highly recommended).
Self-management (22, 23): Overweight and obesity are recognized risk factors for the onset of KOA, which can lead to joint pain and even disability in patients. Diet control combined with exercise can improve the efficacy of weight loss in the treatment of KOA symptoms (Evidence level: Level II, highly recommended).

### Exercise therapy

Knee osteoarthritis patients should practice therapy under the guidance of a doctor, such as straight leg elevation, Tai Chi (23), and Baduanjin (24) (Evidence level: Level I, highly recommended).

#### Description of the evidence

A meta-analysis (23) involving eight randomized controlled trials (RCTs) showed that Tai Chi can improve pain in patients with KOA (ES = −0.75, 95% confidence interval [CI]: −0.99 to −0.51; Q = 8.9, *p* = 0.26; I^2^ = 21%), reduce rigidity (ES = −0.70, 95%CI: −0.95 to 0.46; Q = 9.6, *p* = 0.21; I^2^ = 27%), and improve activity (ES = −0.91, 95%CI: −1.12 to 0.70; Q = 7.2, *p* = 0.40; I^2^ = 3%). A meta-analysis (24) involving seven RCTs showed that Baduanjin could improve KOA patients’ pain (mean difference [MD] = 1.69, 95%CI: 2.03–1.35, *p* < 0.01), reduce rigidity (MD = 0.86, 95% CI: 1.13–0.58, *p* < 0.01), and improve functional activity (MD = 2.23, 95%CI: 3.65–0.82, *p* < 0.01).

### Manual therapy

Manual therapy is used to reduce pain and restore knee motion in KOA patients. The use of pushing and kneading point pressing, pulling and stretching the knees, shaking the knees, and holding and plucking the knees, can play a role in relaxing the tendons and clearing collaterals, promoting blood circulation and removing blood stasis, releasing adhesion, and smoothing the joints, which can improve joint stiffness and muscle strength, reduce joint pain, and improve joint function (25, 26). Patients with infection, skin lesions, tumors, and cardiovascular and cerebrovascular diseases should be treated with caution (Evidence level: Level I, highly recommended).

Manual therapy can effectively relieve the clinical symptoms of patients with KOA and improve their quality of life, without obvious adverse reactions.

#### Description of the evidence

A meta-analysis (25) involving eight RCTs showed that manual therapy improved the healing rate (odds ratio [OR] = 1.81, 95%CI: 1.14–2.88, *p* < 0.01) and the apparent rate for KOA (OR = 2.03, 95%CI: 1.43–2.88, *p* < 0.01), and there were no reports of serious adverse reactions in the included studies. A meta-analysis (26) that included 16 studies showed that manual therapy improved the total response rate (OR = 4.53, 95%CI: 3.06–6.69, *p* < 0.00001), reduced pain (MD = −2.72, 95%CI: −4.19 to −1.25, *p* < 0.00001), improved the WOMAC score (MD = −14.21, 95%CI: −14.86 to −13.56, *p* < 0.00001), and improved the hospital knee joint score (MD = 6.32, 95%CI: 4.58–8.06, *p* < 0.00001).

### Acupuncture

Acupuncture can reduce pain and restore knee motion in KOA patients. Acupuncture, including millimeter acupuncture therapy, warm acupuncture therapy, and electric acupuncture therapy, has a positive effect on relieving KOA pain and improving joint function (27, 28). Moxibustion, which integrates heat therapy, light therapy, drug stimulation, and specific acupoint stimulation, can effectively reduce the vascular permeability of inflammatory foci, improve hemorheologic and hemodynamic indices, relieve knee pain, improve joint function, and improve patients’ quality of life in clinical application (29, 30) (Evidence level: Level I, highly recommended).

#### Recommended acupoints

Xiyan, Yanglingquan, Zusanli, Xuehai, Liangqiu, internal Xiyan, Yinlingquan, Heding, Xiyangguan, and Pian (31).

#### Description of the evidence

A meta-analysis (27) involving 11 RCTs showed that acupuncture was effective in reducing pain in patients with KOA [*n* = 2,387; standard mean difference (SMD) = −0.12, 95%CI: −0.20 to −0.04; I^2^ = 0%] and in improving functional activity [*n* = 2,408; MD = −1.25, 95%CI: −1.97 to −0.53; I^2^ = 0%]. A meta-analysis (28) that included 14 studies showed that compared with Western medicine, warm acupuncture treatment is more effective in improving the long-term overall effective rate [relative risk (RR) = 1.16, 95%CI: 1.04–1.29, *p* = 0.008] and the short-term cure rate (RR = 2.35, 95%CI: 1.59–3.45, *p* < 0.0001), with fewer adverse reactions than Western drugs (RR = 0.20, 95%CI: 0.05–0.75, *p* = 0.02). A meta-analysis (29) of 13 studies showed that moxibustion was superior to conventional care plus sham moxibustion in reducing WOMAC scores (MD = 7.56, 95%CI: 4.11–11.00, *p* = 0.00). Most of the adverse events caused by moxibustion can be cured without medical treatment. An overview (involving a re-meta-analysis) (30) that included 10 systematic reviews showed that moxibustion and moxibustion combined therapy improved the overall response rate in KOA patients (RR = 1.17, 95%CI: 1.13–1.21, *p* < 0.001). Four studies reported 10 common discomfort symptoms caused by moxibustion; these adverse events can be resolved naturally, or even avoided, so moxibustion is safe in the treatment of KOA.

### Acupotomy

Patients with KOA with confirmed or highly suspected soft tissue adhesion can be treated with acupotomy. Acupotomy can be performed on the suprapatellar bursa, subpatellar fat pad, internal Xiyan-acupoint, external Xiyan-acupoint, tibial collateral ligament, iliotibial bundle, and anseropodium sac. By cutting, separating, spatulating, adjusting, and releasing tendon ligament and other soft tissues, the biomechanical balance of the knee joint can be achieved. It is suitable for KOA patients with knee pain, morning stiffness, muscle adhesion, functional limitation, and obvious contracture flexion deformity. It can relieve knee pain and improve joint function, with good safety (32) (Evidence level: Level II, highly recommended).

Description of the evidence: A meta-analysis (32) including 20 RCTs showed that the effective treatment rate in the acupotomy group was higher than that in the acupuncture group (χ^2^ = 11.920, *p* = 0.610, I^2^ = 0%, RR = 1.16, 95%CI: 1.11–1.22). The VAS score of the acupotomy group was lower than that of the acupuncture group (χ^2^ = 94.340, *p* = 0.000, I^2^ = 89%, MD = −1.24, 95%CI: −1.58 to −0.90, *p* = 0.000). In conclusion, the available evidence shows that acupotomy is an effective method for the treatment of KOA, and the efficacy is higher than that of acupuncture therapy.

### Physical therapy

Physical therapy is recommended to help patients with KOA with related symptoms. Common methods include thermal therapy (33), magnetic therapy (34), infrared irradiation (35), hydrotherapy (36), wax therapy (37), ultrasound (38), and other physiotherapy methods, which can be combined with acupuncture, manipulation, and other therapies to improve joint activity, relieve pain and muscle tension, promote local blood circulation, and reduce inflammatory (Evidence level: Level I, Highly recommended).

### Braces

Braces are recommended for patients with mobility difficulties or KOA patients with patellar arthritis. They have the following purposes:

① Reduce the load on the affected joints: Canes and walkers can be used to assist activities (39).
② Joint protection: Elastic sleeves can be worn to protect joints, such as knee pads. The treatment of patellofemoral compartment osteoarthritis with a patellofemoral medial patellofemoral patch can significantly reduce pain. Medial ventricular osteoarthritis of the knee joint can be assisted by wedge insoles (40–42) (Evidence level: Level I, weak recommendation).

### Drug treatment

Sometimes, non-drug treatment is ineffective, and drug treatment can be selected according to the condition of joint pain.

### Drugs for external use

Knee osteoarthritis patients can use TCM, proprietary Chinese medicines, or non-steroidal anti-inflammatory drugs (NSAIDs) for topical use:

① The topical use of Chinese herbs mainly includes fumigation, application, and iontophoresis (43–45) (Level of evidence: I, highly recommended).
② The external use of proprietary Chinese medicines mainly includes various pastes, plagues, ointments, and tinctures (46–48) (Level of evidence: I, highly recommended).
③ Non-steroidal anti-inflammatory preparations for local topical use, which may cause minor adverse reactions, can reduce joint pain and tenderness (49) (Evidence level: Level II, highly recommended).

### Joint cavity injection therapy

Joint viscoelastic supplement therapy with agents such as sodium hyaluronate and medical chitose (joint cavity injection) should be used according to doctors’ clinical experience and patients’ specific conditions (50–52). Platelet-rich plasma is rich in a variety of growth factors and inflammatory regulators, which can protect chondrocytes, promote cartilage healing, reduce intra-joint inflammation, relieve pain, and improve joint function (53). Injection of long-acting corticosteroids into the joint can relieve pain and reduce exudation. The curative effect lasts for several weeks to several months, and repeated injection in the same joint is preferred to avoid aggravating articular cartilage; the injection interval should not be shorter than 4–6 months (2, 54).

Description of the evidence: a meta-analysis (50) of 89 studies showed that joint viscoelastic supplements reduced pain symptoms in patients but increased the risk of adverse events. Results of an RCT (51) involving 82 patients showed no difference in Knee Society Score (KSS) function and VAS scores between the sodium hyaluronate group and the steroid group at 4 weeks, and sodium hyaluronate was significantly better than the steroid at 6 months. A meta-analysis (52) of 54 studies showed that intra-articular injection of sodium hyaluronate could provide relief within 4 weeks. Patients with joint pain symptoms reached a peak response within 8 weeks, and the treatment effect was superior to acetaminophen painkillers, NSAIDs, and COX-2 inhibitors. Results of a meta-analysis that included 15 studies (53) showed that in terms of long-term pain relief and functional improvement of the knee joint, platelet-rich plasma (PRP) injection may be more effective than sodium hyaluronate injection, but the optimal dose, the time interval and frequency of injections, and the ideal treatment for the different stages of KOA remain uncertain. In an RCT (54) that included 66 patients, before treatment and at 7 and 28 days after treatment, the WOMAC scores were 99.6 ± 38.9, 44.2 ± 23.5, and 25.4 ± 21.5, respectively; the treatment of KOA with joint injection of semetasone palmitate can improve the pain symptoms, cystic effusion, and inflammation, with few adverse reactions.

### Diagnosis and prescription for Chinese medicine

#### Chinese medicine

##### Dampness-cold obstruction syndrome

Treatment is based on the principles of warm channel dispelling cold, nourishing blood, and activating the pulse-beat (14, 55–59).

Main prescription: Modified Juanbi Decoction (*The Golden Mirror of Medicine*; Highly recommended).

Common drugs: *Notopterygium incisum* Ting ex H. T. Chang, *Saposhnikovia divaricata* (Trucz.) Schischk, *Angelica sinensis* (Oliv.) Diels, Glycyrrhizae, Paeonia tacti lora Pall, and *Zingiber officinale* Roscoe.

##### Dampness-heat obstruction syndrome

Treatment is based on clearing heat dehumidification, clearing collaterals, and relieving pain (14, 60).

Main prescription: Modified Simiao Decoction (*Danxi’s Experiential Therapy*) (Weak recommendation).

Common drugs: *Atractylodes lancea*, *Coix lacryma-jobi*, *Achyranthes bidentata* Blume, *Lonicera japonica* Thunb, *Trachelospermum jasminoides* Lindl. Lem, and *Phryma leptostachya* L. subsp. *asiatica* (Hara) Kitamura.

##### Qi-blood stagnation syndrome

Treatment is based on promoting blood circulation, removing blood stasis, clearing collaterals, and relieving pain (14, 61).

Main prescription: Modified Taohong Siwu Decoction (*YiLei YuanRong*; Highly recommended).

Common drugs: *Rehmannia glutinosa* (Gaetn.) Libosch. ex Fisch. et Mey, *Angelica sinensis* (Oliv.) Diels, Paeonia tacti lora Pall, *Ligusticum chuanxiong* hort, *Prunus persica* (L.) Batsch, and *Carthamus tinctorius* L.

##### Deficiency of liver and kidney

Treatment is based on nourishing liver and kidney (14, 62).

Main prescription: Modified Duhuo Jisheng Decoction (*Precious Essential Formulary for Emergency*; Weak recommendation).

Common drugs: *Taxillus sutchuenensis* (Lecomte) Danser, *Eucommia ulmoides* Oliver, *Achyranthes bidentata* Blume, *Asarum sieboldii* Miq, *Poria*, and *Gentiana macrophylla*.

##### Qi-blood weakness syndrome

Treatment is based on supplementing qi and nourishing blood (14, 63).

Main prescription: Modified Bazhen Decoction (Danxi’s Experiential Therapy; Highly recommended).

Common drugs: *Panax ginseng* C. A. Mey, *Cinnamomum cassia* Presl, *Ligusticum chuanxiong* hort, *Rehmannia glutinosa* (Gaetn.) Libosch. ex Fisch. et Mey, *Poria*, *Angelica sinensis* (Oliv.) Diels, and Paeonia tacti lora Pall.

### Chinese patent medicine

It is recommended that clinicians give proprietary Chinese medicines (PCMs) to KOA patients according to their TCM syndrome differentiation. There are various kinds of PCM for the treatment of KOA. Biqi capsule, Longbii capsule, Huli San capsule, Xianling Gubao capsule, Jintiange capsule, and Zhuanggu Guanjie capsule (61–65) can be selected (Evidence level: Level I, weak recommendation).

Description of the evidence: a meta-analysis (64) involving 12 RCTs showed that Huli San capsule treatment improved the symptom remission rate of KOA (RR = 1.38, 95%CI: 1.13–1.69, *p* = 0.02) and the knee function score (MD = 2.88, 95%CI: 0.81–4.94, *p* = 0.006) compared to conventional treatment. There was no significant difference in VAS score (MD = −0.57, 95%CI: −1.42 to −0.29, *p* = 0.19). The results showed that the use of Huli San capsule or Huli San capsule plus conventional Western medicine treatment could improve the symptom relief rate, Lysholm score, knee joint function score, and VAS score of patients with KOA and alleviate the symptoms of knee joint pain, swelling, and movement restriction; moreover, no serious adverse reactions have been reported. A meta-analysis (65) that included seven studies showed that compared with the control group, the treatment response rate in the experimental group was improved (OR = 4.63, 95%CI: 2.83–7.56, *p* < 0.01). The WOMAC score of the test group was lower than that of the control group [weighted mean difference (WMD) = −13.14, 95%CI: −22.07 to −4.22, *p* < 0.05]. The VAS score of the test group was lower than that of the control group (WMD = −15.49, 95%CI: −18.84 to −12.15, *p* < 0.01). There was no significant difference in the incidence of adverse reactions between the experimental group and the control group (OR = 0.73, 95%CI: 0.30–1.78, *p* > 0.05). In conclusion, Zhuanggu Guangjie capsule can reduce the VAS score and WOMAC score of patients with KOA and improve the effective rate, and the clinical efficacy is good, but the risk of adverse reactions should be paid attention to.

A network meta-analysis (66) involving 58 RCTs showed that the top three interventions with the best total response rate were Jinwu Gutong capsule + Glucosamine (GlcN), Xianling Gubao + aminosaccharide, and Biqi capsule. The top three interventions with the best VAS score were Panlongqi tablet, Xianling Gubao + GlcN, and Xianling Gubao + NSAIDs. The top three interventions with the best reduction of WOMAC total score were Jintiange capsule + NSAIDs, Jinwu Gutong capsule + GlcN, and Biqi capsule + NSAIDs. The three interventions with the best efficacy in reducing the Lequesne index were Xianling Gubao + NSAIDs, Biqi capsule + NSAIDs, and Jintiange capsule + NSAIDs. The three best interventions to reduce the level of TNF-α were Xianling Gubao + GlcN, Jintiange capsule, and Jintiange capsule + GlcN = Jinwu Gutong capsule + GlcN. In terms of safety, the top five interventions with the fewest adverse reactions were Biqi capsule, Jinwu Gutong capsule, Biqi capsule + NSAIDs, Xianling Gubao + NSAIDs, and Jintiange capsule. The results showed that the combination of PCM and NSAIDs or GlcN could improve the clinical therapeutic effect and reduce the adverse reactions in patients with KOA.

A meta-analysis (67) of 21 RCTs including 16 papers using the effective rate of continuous variables as the index showed that the Xianling Gubao group was superior to the control group in terms of effective rate (RR = 1.21 95%CI: 1.16–1.26, *p* < 0.00001). A total of five studies were included with bicategorical variables as the index of pain relief time. Xianling Gubao was superior to the control in shortening pain relief time (MD = −1.51, 95%CI: −1.81 to −1.21, *p* < 0.00001). A total of five studies were included using bicategorical variables to improve the Lysholm knee function score, and Xianling Gubao could significantly improve knee function compared with the control group (MD = 17.21 95%CI: 10.02–24.39, *p* < 0.00001). In addition, adverse reactions were reported in five of the 21 included studies, all of which resolved spontaneously without specific treatment. In conclusion, Xianling Gubao capsule can effectively improve several indices of patients with KOA and is worthy of clinical promotion and use.

A meta-analysis (68) including six RCTs showed that Biqi capsule combined with Western medicine resulted in a better overall response rate in the treatment of KOA than Western medicine alone (RR = 1.22, 95%CI: 1.15–1.29, *p* < 0.00001), and the WOMAC score was lower than that of the Western medicine group (MD = −10.57, 95%CI: −12.17 to −8.97, *p* < 0.00001). Biqi capsule combined with Western medicine was better than Western medicine alone in reducing the VAS score of KOA patients and improving their quality of life, and no serious adverse events occurred. In conclusion, Biqi capsule combined with Western medicine has good clinical effects and safety in the treatment of KOA.

### Pain relief drugs

Medications can be taken orally to relieve pain in KOA patients:

① Acetaminophen: Because the elderly are prone to adverse reactions to NSAIDs and synovitis is not a major factor in the early stage of KOA, short-term acetaminophen can be used in mild cases (69).
② NSAIDs: These have both analgesic and anti-inflammatory effects and are the most commonly used drugs to control osteoarthritis symptoms (70). The main adverse effects include gastrointestinal symptoms, kidney or liver functional impairment, impaired platelet function, and an increased risk of cardiovascular adverse events. They should be used with caution if the patient is at risk for cardiovascular adverse events.
③ Opioids: For patients with acute pain attacks, when acetaminophen and NSAIDs cannot fully relieve pain or there is a drug contraindication, weak opioids can be considered, which are better tolerated and less addictive, such as oral tramadol. Such treatments should be started at a low dose and slowly increased every few days to reduce adverse reactions (71) (Evidence level: Level I, highly recommended).

### Disease-modifying OA drugs and cartilage protectants

Such drugs tend to work slowly, taking weeks of treatment to exert effects. Therefore, they are called slow acting drugs. They can reduce the activity of matrix metalloproteinase and collagenase, and they can not only exert anti-inflammatory and analgesic effects but can also protect articular cartilage and delay the development of KOA. However, there is no recognized ideal drug at present, and commonly used drugs such as GlcN, Diacerein, and chondroitin sulfate may have certain effects (72–74) (Evidence level: Level I, highly recommended).

### Surgery

For patients with recurrent knee swelling pain and joint effusion, the effect of non-surgical treatment is not good, the pain is progressive, the joint is obviously dysfunctional, and surgical treatment can be considered to correct the deformity and improve joint function. Surgical treatment is recommended after assessment of the condition and surgical indications.

### Arthroscopic surgery (level of evidence: I)

Arthroscopic surgery is recommended for early and middle stage patients (75–78). Arthroscopy is both diagnostic and therapeutic, mainly for patients with mechanical locking or meniscal tears and other symptoms. Through arthroscopic free body cleaning and meniscus molding, the symptoms of some patients in the early and middle stages can be alleviated, the internal joint environment can be improved, and the synovial inflammatory reaction can be alleviated. For advanced patients with abnormal force lines and obvious osteophytic hyperplasia, arthroscopic irrigation or clearance alone has poor results.

Description of the evidence: a meta-analysis (75) of nine studies showed that pain symptoms in patients with KOA could be improved at 3 and 6 months compared with the control group, but there was no significant improvement in motor function.

An RCT (76) that included 107 patients showed that the average WOMAC scores of 56 patients in the operation group were 624 ± 98 and 865 ± 589 at 1- and 2-year follow-up, respectively. The average WOMAC scores of 51 patients in the control group were 902 ± 521 and 914 ± 605 at 1- and 2-year follow-up, respectively. At 1-year follow-up, the WOMAC score of the operation group was significantly different from that of the control group (*p* < 0.05), but there was no significant difference in WOMAC score between the two groups at 2 years after surgery (*p* > 0.05). In conclusion, limited arthroscopic surgery can provide short-term (≤1 year) symptom relief in patients with KOA.

An RCT (77) involving 70 patients showed that the VAS score decreased by 24.45 ± 9.09, 18.45 ± 11.60, and 8.29 ± 14.19% in the PRP treatment group at 3, 6, and 9 months, respectively. In the arthroscopic treatment group, the VAS score decreased by 18.96 ± 5.85, 7.33 ± 8.60, and 3.20 ± 7.39% at 3, 6, and 9 months, respectively. A statistically significant difference was observed between the two groups only at 3 and 6 months (*p* < 0.05). The WOMAC score decreased by 24.03 ± 11.41, 17.45 ± 9.24, and 9.49 ± 9.80% in the PRP group and by 11.27 ± 5.73, 5.70 ± 4.78, and 0.13 ± 5.06% in the arthroscopic group at 3, 6, and 9 months, respectively. At all three time points, the difference between the two groups was statistically significant (*p* < 0.001). In conclusion, this study suggests that both PRP and arthroscopy can reduce the WOMAC and VAS pain scores in patients with KOA.

### Periknee osteotomy (evidence level: level I)

Periknee osteotomy is suitable for younger (generally <65 years old), relatively active patients with good bone mass, external joint deformity, and meniscus protrusion. The knee joint structure is preserved to the maximum extent, and KOA symptoms are relieved by changing the lower limb force line, improving function and effectively relieving joint pain in patients.

1. Proximal tibial osteotomy: The tibia is the main varus deformity, with MPTA <85°. Medial KOA meets the following characteristics: ① grade 0 cartilage wear: young active patients with obvious varus deformity, symptoms after exercise, and a strong desire for surgery; ② meniscus injury period and partial cartilage wear period: it is a strong indication period, especially for patients over 45 years old with imaging manifestations of internal and lateral ventricular hypertension and ineffective conservative treatment; ③ bone contact stage: patients with active exercise and significant proximal varus deformity or patients who refuse to undergo joint replacement and have good mobility and are not obese (79–83).
2. Distal femoral osteotomy: Distal femoral osteotomy is performed in patients with distal femoral malformations. It is mainly used in patients with lateral interventricular KOA with valgus deformity (84, 85) or medial interventricular KOA with distal femoral deformity.

Description of the evidence: a meta-analysis (79) involving 13 studies showed that medial tibial open wedge osteotomy and lateral tibial closed wedge osteotomy were mainly associated with postoperative posterior inclination of the tibial plateau (MD = 2.82, 95%CI: 1.31–4.33, *p* = 0.0002). Significant differences in patellar height BPI (MD = −0.09, 95%CI: −0.11 to −0.07, *p* < 0.00001) and operative time (MD = −19.48, 95%CI: −31.02 to −7.94, *p* = 0.0009) were also found. Both surgical methods had similar effects on the postoperative mechanical axis angle (MD = −0.01, 95%CI: −0.51 to 0.48, *p* = 0.96), correction angle (MD = −0.16, 95%CI: −0.75 to 0.43, *p* = 0.60), HSS score (MD = −0.46, 95%CI: −1.47 to 0.55, *p* = 0.37), VAS score (MD = 0.12, 95%CI: −0.24 to 0.48, *p* = 0.51), Lysholm score (MD = −0.17, 95%CI: −2.53 to 2.19, *p* = 0.89), and complications (OR = 0.68, 95%CI: 0.25–1.82, *p* = 0.44). In conclusion, the overall clinical efficacy of medial open tibia and lateral closed wedge osteotomy in the treatment of single compartment KOA is similar, but the medial open wedge osteotomy is easier to perform, and it is easy to increase the posterior inclination of the tibial plateau and the patella decline after surgery. The clinician should conduct adequate preoperative imaging evaluation and individual selection of the appropriate surgical procedure for patients with single compartment KOA.

A meta-analysis (80) that included five studies showed that there was no significant difference between the experimental group and the control group in terms of bone non-union.

Alternative materials combined with locking plate technology provide a safe and effective alternative to open wedge high tibial osteotomy (HTO) with an osteotomy gap greater than 10 mm, but there is a possibility of bone non-union.

A meta-analysis (81) that included 19 studies and a meta-analysis (82) that included 11 studies showed that HTO was superior to monocondylar replacement in terms of range of motion.

The results of a clinical study (84) that included 22 patients showed that the femoral tibial angle, negative gravity line ratio, and distal lateral femur angle were significantly improved 1 year after surgery compared with those before surgery (*p* < 0.05). The pain VAS score and knee HSS score were significantly improved at 1 and 2 years after surgery (*p* < 0.05), but there was no significant difference between these scores at 1 year after surgery and these scores at 2 years after surgery (*p* > 0.05). In conclusion, distal medial wedge osteotomy of the femur is an ideal knee-saving treatment method for patients with osteoarthritis combined with knee valvaration. It can achieve satisfactory medium-term clinical results and is beneficial to the repair of lateral interventricular cartilage of the knee joint.

A clinical study (85) that included 33 patients showed that the HSS score was increased after surgery (*p* < 0.05), while the VAS score was decreased (*p* < 0.05). Imaging examinations showed that the positions of the femoral tibial angle (aFTA), distal lateral angle of the femur (aLDFA), and mechanical axis in the tibial plateau were significantly improved after operation (*p* < 0.05). All the bones healed. In conclusion, distal medial osteotomy of the femur can effectively correct the line of force of lower limbs, reduce the pressure load of the lateral compartment, and preserve the original motion of the knee joint, with accurate clinical effects.

A clinical study (86) involving 15 patients showed that there were 14 cases of bone healing at the osteotomy 3 months after surgery, and one case of bone healing was delayed until 6 months after surgery due to fracture of the bone cortex at the osteotomy hinge during surgery. There was no significant difference in knee flexion motion and Kellgren–Lawrence grade of osteoarthritis 2 years after surgery (*p* > 0.05). The knee valgus angle was significantly corrected 3 months after surgery, and the KOOS score of knee function was significantly increased 2 years after surgery (*p* < 0.05). In conclusion, distal medial femoral closure osteotomy is effective in the treatment of lateral interventricular osteoarthritis of the knee. Postoperative complications of osteotomy non-union and internal fixation stimulation are less common, and the operation does not affect knee flexion mobility. Patients can exercise with early weight bearing function.

### Unicompartmental knee arthroplasty (level of evidence: level I)

Patients with severe unilateral knee wear or abnormal alignment can be treated with unicompartmental knee arthroplasty (UKA) (87–89). UKA includes medial and lateral monocondylic replacement. Medial single condylar replacement is suitable for patients with single compartment osteoarthritis of the knee whose medial joint wear is dominant, the force line is changed by 5–10°, the ligament is intact, and the flexion contracture is not more than 15°. Lateral single condylar replacement is suitable for bone-to-bone knee lateral compartment osteoarthritis with normal medial compartment cartilage, flexion and valgus deformity <15°, flexion and extension motion >90°, intact anterior cruciate ligament, normal function of the posterior cruciate ligament, and stable and correctable valgus deformity of the knee joint. UKA preserves the normal structure of the knee as much as possible in order to obtain better proprioception and functional recovery. Description of the evidence: A meta-analysis that included 17 studies (87) showed that the postoperative complications (OR = 4.52, 95%CI: 2.30–8.90, *p* < 0.001), Lysholm score (MD = −5.53, 95%CI: −11.11 to 0.05, *p* = 0.05), and revision rate (OR = 1.67, 95%CI: 1.01–2.76, *p* = 0.05) were significantly better in the UKA group than in the HTO group. There were no significant differences between the two groups in operation time, blood loss, other knee function scores, range of joint motion, rate of clinical outcome, force of lower limb line, and cartilage degeneration (*p* > 0.05). In conclusion, HTO and UKA can achieve similar and satisfactory clinical outcomes in the treatment of medial ventricular osteoarthritis of the knee. In contrast, UKA is superior to HTO in terms of postoperative complications, Lysholm score, and revision rate.

A meta-analysis of 24 studies showed that (88) the intraoperative blood loss (*p* < 0.05), drainage volume (*p* < 0.05), blood transfusion rate (*p* < 0.05), operation time (*p* < 0.05), KSS score (*p* < 0.05), HSS score (*p* < 0.05), and knee motion (*p* < 0.05) in the UKA group were better than those in the total knee replacement group, but the renovation rate of the former was significantly higher than that of the latter (*p* < 0.05). There was no significant difference in postoperative complications or in the improvement in curative effect (*p* > 0.05). In conclusion, UKA is beneficial to reduce intraoperative blood loss, drainage volume, blood transfusion rate, and operation time, and it improves the knee joint score and range of motion. The advantage of total knee replacement is a lower revision rate. Clinical planning for KOA patients should pay more attention to their own conditions and needs.

### Total knee arthroplasty (level of evidence: I)

Total knee arthroplasty (TKA) is suitable for the treatment of severe multi-ventricular osteoarthritis of the knee joint, especially with severe joint pain and deformity, and the daily life of patients with severe symptoms is strongly affected after non-surgical treatment; it is also suitable for KOA patients after receiving a failed orthopedic osteotomy (90–93). TKA is effective in relieving pain and improving joint function.

Description of the evidence: a meta-analysis of 191 studies (90) showed that patients receiving primary TKA experienced rapid improvements in pain and function during the first year after surgery. After 10 years, pain might not be alleviated, but function has improved.

A meta-analysis of 19 studies (91) showed that most patients were satisfied with the procedure and their daily functional activities had benefited significantly. TKA resulted in significant medium- and long-term outcomes in terms of pain and function, resulting in high patient satisfaction.

Results of a clinical study (92) involving 95 patients showed that in patients with KOA eligible for unilateral TKA, non-surgical treatment after TKA provided better pain relief and improved function after 12 months than non-surgical treatment alone.

## Prevention and care

### Prevention (highly recommended)

Patients with KOA should pay attention to appropriate prevention and care. The main prevention methods are as follows:

① Strict control of weight and diet. Weight reduction is very beneficial to reduce the joint burden, improve joint function, reduce pain, and so on (2, 9).
② Reducing the trauma of the knee joint. Patients should reduce the trauma of the knee joint and avoid repeated stress.
③ Preventing osteoporosis, often participating in outdoor activities, getting more sunshine, and so on. Patients with severe osteoporosis should be given anti-osteoporosis therapy.
④ Doing correct exercises and avoiding strenuous activities, such as long-distance running, repeated squatting, kneeling, and lifting heavy objects.

### Care (highly recommended)

① Patients should pay attention to the changes of the four solar terms, including the wind, cold, and summer humidity.
② Patients should avoid standing and walking for a long time and pay attention to knee joint protection.
③ Proper rest and the use of canes can reduce the load on the affected joints.
④ Aerobic activities such as lifting legs and stretching knees in bed, walking, swimming, and cycling help maintain joint function (2, 10).
⑤ Suitable shoes and insoles can absorb shocks.

## Summary and conclusion

Patients with KOA should be treated in stages and steps, and patients at different stages of the disease may be treated in roughly the same way, but the focus should be different. For patients with stage I (pre-stage) symptoms, a clear diagnosis should be made, attention should be paid to the health education of the diagnosed patients, and the awareness of prevention and care should be strengthened. Risk factors should be controlled, such as controlling blood sugar, choosing appropriate shoes, reducing excessive weight bearing of the knee, and performing exercise. Patients whose lives are seriously impacted can consider therapy and TCM treatment, such as acupuncture, manual therapy, or electroacupuncture. Premature drug treatment is not recommended.

In stage II (early stage) and III (middle stage) KOA patients, the focus is on avoiding artificial joint replacement surgery and using the patient’s original joint as much as possible, but surgery can be considered to improve the patient’s knee deformity. For patients diagnosed with stage II (early stage) KOA, osteotomy and orthosis can be selected according to the patient’s knee condition on the basis of health education, strengthening prevention and care, and non-drug treatment (exercise, therapy, and TCM). For patients with stage III (middle stage) KOA, drugs can be used to control pain and other symptoms. For example, TCM compounds can be used in combination with Western medicine to help patients reduce the frequency of taking Western medicine and regulate the patients’ Zangfu function. Meanwhile, acupotomy and other means can be used to help restore knee motion. Orthopedic osteotomy may be considered for patients with significant deformities. However, patients should also be properly informed that the relevant symptoms are not easy to control, and psychological counseling should be given.

For patients with stage IV (late stage) KOA, TKA surgery should be avoided as far as possible. If the combination of drug therapy and non-drug therapy does not achieve satisfactory clinical results, knee saving surgery (arthroscopic debridement, HTO, UKA) can be considered.

For patients with stage V (surgery stage) KOA, it is recommended to perform TKA, focusing on the prevention of relevant complications while actively carrying out rehabilitation exercise, and after surgery, drug therapy or non-drug therapy can be given to actively help patients recover, so that patients can return to society faster.

This study has some limitations. First, the most important primary screening information in the diagnosis of KOA is the physical examination data, radiographic features, and the patient’s complaints. For example, the combination of osteophytes and knee pain is known to be a very important clue for the diagnosis of KOA, with a likelihood ratio of more than 10. It may be helpful for clinicians to make an accurate diagnosis if the guideline provides information on the relationship between specific diagnostic test results and the patient’s physical examination, sensitivity, and specificity, along with citations to previous studies. It should provide more detailed information about valuable physical examinations that function better as high-quality information for screening diagnosis. Second, there are three internationally recognized clinical classification criteria for KOA: the European League Against Rheumatism (EULAR), the American College of Rheumatology (ACR), and the National Institute for Health and Care Excellence (NICE) criteria. Although the references for the criteria presented in the Diagnostic criteria section of this manuscript are from China, they are considered to be based on the ACR criteria in terms of content. For the completeness of the guideline, it should further present and compare at least two of the above criteria from outside China, such as EULAR and NICE, and the current guideline based on the references mentioned in this manuscript in the next step. Third, for the clinical staging and Chinese classification of KOA, we think that the “theory of Chinese treatment of non-disease” is a concept that should be considered in modern KOA treatment. However, unfortunately, it seems difficult to find previous epidemiological studies that support the classification. As the latest KOA-related research supports the concept of prevention before the onset of disease, more studies are needed, for example, actively incorporating relevant pathophysiological information into this guideline. It would be helpful if the guidelines provide as much updated evidence as possible to support the staged treatment of KOA. For KOA-related interventions such as acupuncture and Tai Chi, further research on standardized practices is needed. Additional information about the dosage and duration of each remedy needs to be obtained. There may be interaction or safety issues when TCMs/PCMs are used together with Western synthetic drugs, which need to be further explored. Finally, the manner in which the KOA diagnosis is made requires greater detail. For instance, is there an initial screening questionnaire? How were radiographic findings standardized? Were there any other tools or assessment methods, such as gait analysis, that were used to support the diagnosis? Theoretically, the proposed criteria seem robust, but their true utility in KOA diagnosis will only be clarified in real-world settings. These criteria should be tested in various clinical environments, including primary care settings. These issues show that more in-depth research is necessary.

To sum up, for patients with confirmed KOA, we should consider the evidence-based staging and stepped treatment of KOA and provide TCM treatment to improve clinical outcomes and extend the service life of the joint. Clinical treatment should be based on multiple approaches, and attention should be paid to the physical and mental health of patients.

## Evidence quality classification

First, according to the type of study, the quality of the included studies was evaluated using a scale. Studies that meet the requirements (score ≥ 5 on the AMSTAR scale for meta-analyses, score ≥ 3 on the modified Jadad scale for RCTs, score ≥ 13 for non-randomized clinical trials) were classified according to the criteria in Table 5. The final quality of evidence was graded according to the recommendation grade standard of TCM literature (Table 5).

**Table 5:** TCM literature basis and recommendation level.

TCM literature classification		TCM literature recommended grade	
Level I	Large sample randomized controlled trials with clear results and low false positive or false negative rates	Grade I	Supported by at least two Level I studies
Level II	Small sample randomized controlled trials with uncertain results and high false positive or false negative rates	Grade II	Supported by only one Level I study
Level III	Non-randomized, contemporaneous controlled trials based on expert consensus from ancient literature	Grade III	Supported only by Level II studies
Level IV	Historical comparative study, contemporary expert consensus	Grade IV	Supported by at least one Level III study
Level V	X-ray (standing or weight-bearing position), case reports, uncontrolled studies, expert opinions, and papers on osteophyte formation at the joint margin	Grade V	Supported only by Level IV or Level V studies

## Recommended strength classification

The nominal group method was used to grade the recommendation strength, and experts voted based on factors such as evidence level, efficacy, safety, economy, and patient acceptance. The recommended direction includes recommended and not recommended, and the recommended strength includes strong and weak.

## Author contributions

LZ: Project administration, Writing – original draft. GZ: Formal analysis, Software, Writing – original draft. WY: Writing – review & editing, Methodology, Validation. JL: Writing – review & editing, Supervision.

## Group members of China Association for Chinese Medicine

Jianke Pan, The Second Affiliated Hospital of Guangzhou University of Chinese Medicine; Yanhong Han, The Second Affiliated Hospital of Guangzhou University of Chinese Medicine; Hetao Haung, The Second Affiliated Hospital of Guangzhou University of Chinese Medicine; Houran Cao, The Second Affiliated Hospital of Guangzhou University of Chinese Medicine; Desheng Chen, Tianjin Hospital; Dingjia Chen, Zhangzhou Traditional Chinese Medicine Hospital; Feng Chen, Ruikang Hospital Affiliated to Guangxi University of Chinese Medicine; Guangxinag Chen, Suzhou Municipal Hospital; Hongyun Chen, The Second Affiliated Hospital of Guangzhou University of Chinese Medicine; Rui Fang, Xinjiang Uygur Autonomous Region Hospital of Traditional Chinese Medicine; Da Guo, The Second Affiliated Hospital of Guangzhou University of Chinese Medicine; Jianying He, Jiangxi Provincial People’s Hospital; Kunhao Hong, Guangdong Second Traditional Chinese Medicine Hospital; Ye Huang, Beijing Jishuitan Hospital; Yongming Huang, The Second Affiliated Hospital of Guangzhou University of Chinese Medicine; Yinghua Lai, The Second Affiliated Hospital of Guangzhou University of Chinese Medicine; Gang Li, Shandong Provincial Hospital of Traditional Chinese Medicine; Yikai Li, Nanfang Hospital; Guihong Liang, The Second Affiliated Hospital of Guangzhou University of Chinese Medicine; Dingkun Lin, The Second Affiliated Hospital of Guangzhou University of Chinese Medicine; Fangzheng Lin, The Second Affiliated Hospital of Guangzhou University of Chinese Medicine; Aifeng Liu, The First Teaching Hospital of Tianjin University of Chinese Medicine; Guobin Liu, The First Hospital of Hebei Medical University; Jianping Liu, Beijing University of Chinese Medicine; Peilai Liu, Qilu Hospital of Shandong University; Wengang Liu, Guangdong Second Traditional Chinese Medicine Hospital; Jian Liu, Beijing Jishuitan Hospital; Minghui Luo, The Second Affiliated Hospital of Guangzhou University of Chinese Medicine; Yuanchen Ma, Guangdong Provincial People’s Hospital; Aihua Ou, The Second Affiliated Hospital of Guangzhou University of Chinese Medicine; Feng Qiao, Honghui Hospital; Puyi Sheng, The First Affiliated Hospital, Sun Yat-sen University; Xin Tang, West China Hospital, Sichuan University; Guangji Wang, Hainan General Hospital; Peimin Wang, Jiangsu Provincial Hospital of Chinese Medicine; Wulian Wang, Fuzhou Second Hospital; Xu Wei, Wang Jing Hospital of China Academy of Chinese Medical Science; Nanjun Xu, The Second Affiliated Hospital of Guangzhou University of Chinese Medicine; Puwei Yuan, Affiliated Hospital of Shaanxi University of Chinese Medicine; Hongsheng Zhan, Shuguang Hospital, Shanghai University of Traditional Chinese Medicine; Min Zhang, Second Hospital of Shanxi Medical University; Di Zhao, The Second Affiliated Hospital of Guangzhou University of Chinese Medicine; Jinlong Zhao, The Second Affiliated Hospital of Guangzhou University of Chinese Medicine; Lilian Zhao, Foshan Hospital of Traditional Chinese Medicine; Xiaofei Zheng, The First Affiliated Hospital of Jinan University; Mingwang Zhou, Gansu Provincial Hospital of Traditional Chinese Medicine; Liguo Zhu, Wang Jing Hospital of China Academy of Chinese Medical Science.

## Figures and Tables

**Table:** 

TCM	Traditional Chinese medicine
KOA	Knee osteoarthritis
Knee osteoarthritis	“Bone impediment (骨痹)” and “bi syndrome”
Knee osteoarthritis	A chronic joint disease characterized by articular cartilage degeneration, changes in subchondral bone reactivity, osteophyte formation at joint edges, synovial disease, ligament relaxation or contracture, capsular contracture, and muscle weakness (1–3)

## References

[1] Sharma L. Osteoarthritis of the knee. N Engl J Med. (2021) 384:51–9. doi: 10.1056/NEJMcp1903768 33406330

[2] Joint Surgery Group, Society of Osteology, Chinese Medical Association. Osteoarthritis treatment guide (2018 edition). Chin J Orthopaed. (2018) 38:705–15. doi: 10.3760/cma.j.issn.0253-2352.2018.12.001

[3] Xu XM Liu WG Zhan HS Liu G Zhang QW Xu SC. Muscle training rehabilitation treatment of knee arthralgia (knee osteoarthritis) expert consensus. Chin Manipul Rehabil Med (2020). 11:1–4. doi: 10.19787/j.issn.1008-1879.2020.19.001

[4] Thomas AC Sowers M Karvonen-Gutierrez C Palmieri-Smith RM. Lack of quadriceps dysfunction in women with early knee osteoarthritis. J Orthop Res. (2010) 28:595–9. doi: 10.1002/jor.21038, PMID: 19918898

[5] Chun SW Kim KE Jang SN Kim KI Paik NJ Kim KW. Muscle strength is the main associated factor of physical performance in older adults with knee osteoarthritis regardless of radiographic severity. Arch Gerontol Geriatr. (2013) 56:377–82. doi: 10.1016/j.archger.2012.10.013, PMID: 23164547

[6] Wang B, Yu N. Consensus of four-stepladder program of knee osteoarthritis (2018). Chin J Joint Surg (2019) 13:124–130.

[7] Kellgren JH Lawrence JS. Radiological assessment of osteo-arthrosis. Ann Rheum Dis. (1957) 16:494–502. doi: 10.1136/ard.16.4.494, PMID: 13498604 PMC1006995

[8] Recht MP Kramer J Marcelis S Pathria MN Trudell D Haghighi P. Abnormalities of articular cartilage in the knee: analysis of available MR techniques. Radiology. (1993) 187:473–8. doi: 10.1148/radiology.187.2.8475293, PMID: 8475293

[9] Chinese Association of Integrative Medicine. Knee osteoarthritis diagnosis and treatment guide of integrated Chinese and Western medicine. Nat Med J China. (2018) 98:3653–8. doi: 10.3760/cma.j.issn.0376-2491.2018.45.005

[10] Chinese Rheumatology Association. Guidelines of osteoarthritis diagnosis and treatment. Chin J Rheumatol. (2010) 6:416–9. doi: 10.3760/cma.j.issn.1007-7480.2010.06.024

[11] Brophy RH Fillingham YA. AAOS clinical practice guideline summary: Management of Osteoarthritis of the knee (nonarthroplasty), third edition. J Am Acad Orthop Surg. (2022) 30:e721–9. doi: 10.5435/JAAOS-D-21-01233, PMID: 35383651

[12] Liu J Zeng LF Yang WY Liang GH Luo MH Chen HY. Exploration on knee osteoarthritis of evidence-based staging and step-by-step treatment strategy based on TCM great health concepts. Chin J Tradition Chin Med Pharm. (2019) 34:1321–7.

[13] Liu J Huang HT Pan JK Zeng LF Liang GH Yang WY. The development status and prospect of step diagnosis and treatment of knee osteoarthritis with integrated traditional Chinese and Western medicine. Guangdong Med J. (2019) 40:1189–92. doi: 10.13820/j.cnki.gdyx.20185817

[14] Chen W. Guidelines for TCM diagnosis and treatment of knee osteoarthritis (2020 edition). J Tradition Chin Orthoped Traumatol. (2020) 32:1–14.

[15] Chen G. Investigation on expert consultation of Delphi method for TCM syndrome of knee osteoarthritis. Yunnan University of Chinese Medicine, Osteomatology of traditional Chinese medicine. (2013).

[16] China Association of Chinese Medicine. Guidelines for the Diagnosis and Treatment of Common Diseases of Orthopaedics and Traumatology in Traditional Chinese Medicine. Beijing: China press of traditional Chinese medicine (2012).

[17] National Administration of Traditional Chinese Medicine. GB/T 15657-2021. Nanjing: State Administration for Market Regulation and the Standardization Administration of the Country. (2021).

[18] Zheng X. Guiding Principles for Clinical Research of New Chinese Medicine. Beijing: China press of traditional Chinese medicine (2002).

[19] Mahmoudian A Lohmander LS Mobasheri A Englund M Luyten FP. Early symptomatic osteoarthritis of the knee-time for action. Nat Rev Rheumatol. (2021) 17:621–32. doi: 10.1038/s41584-021-00673-4 34465902

[20] Zhang L Fu T Zhang Q Yin R Zhu L He Y. Effects of psychological interventions for patients with osteoarthritis: a systematic review and meta-analysis. Psychol Health Med. (2018) 23:1–17. doi: 10.1080/13548506.2017.1282160, PMID: 28140653

[21] Geenen R Overman CL Christensen R Åsenlöf P Capela S Huisinga KL. EULAR recommendations for the health professional's approach to pain management in inflammatory arthritis and osteoarthritis. Ann Rheum Dis. (2018) 77:797–807. doi: 10.1136/annrheumdis-2017-212662, PMID: 29724726

[22] Hall M Castelein B Wittoek R Calders P van Ginckel A. Diet-induced weight loss alone or combined with exercise in overweight or obese people with knee osteoarthritis: a systematic review and meta-analysis. Semin Arthritis Rheum. (2019) 48:765–77. doi: 10.1016/j.semarthrit.2018.06.005 30072112

[23] Kelley GA Kelley KS Callahan LF. Clinical relevance of tai chi on pain and physical function in adults with knee osteoarthritis: an ancillary meta-analysis of randomized controlled trials. Sci Prog. (2022) 105:003685042210883. doi: 10.1177/00368504221088375 PMC10450487 35379041

[24] Zeng ZP Liu YB Fang J Liu Y Luo J Yang M. Effects of Baduanjin exercise for knee osteoarthritis: a systematic review and meta-analysis. Complement Ther Med. (2020) 48:102279. doi: 10.1016/j.ctim.2019.102279, PMID: 31987253

[25] Zhang H Yuan MJ Sun J Chen SC Dou YM. Therapeutic effect of manual therapy on knee osteoarthritis: a meta-analysis. Hainan Med J (2019). 30:925–929. doi: 10.3969/j.issn.1003-6350.2019.07.032

[26] Pan SQ Li N Liu JP Han XF. The safety and efficacy of manual therapy therapy on knee osteoarthritis: a meta-analysis. J Mod Med Health. (2022) 38:3676–85. doi: 10.3969/j.issn.1009-5519.2022.21.015

[27] Tian H Huang L Sun M Xu G He J Zhou Z. Acupuncture for knee osteoarthritis: a systematic review of randomized clinical trials with Meta-analyses and trial sequential analyses. Biomed Res Int. (2022) 2022:1–15. doi: 10.1155/2022/6561633 PMC9050311 35496051

[28] Chen J Liu A Zhou Q Yu W Guo T Jia Y. Acupuncture for the treatment of knee osteoarthritis: an overview of systematic reviews. Int J Gen Med. (2021) 14:8481–94. doi: 10.2147/IJGM.S342435 34848997 PMC8617312

[29] Song GM Tian X Jin YH Deng YH Zhang H Pang XL. Moxibustion is an alternative in treating knee osteoarthritis: the evidence from systematic review and meta-analysis. Medicine (Baltimore). (2016) 95:e2790. doi: 10.1097/MD.0000000000002790, PMID: 26871839 PMC4753935

[30] Yin S Zhu F Li Z Che D Li L Feng J. An overview of systematic reviews of moxibustion for knee osteoarthritis. Front Physiol. (2022) 13:822953. doi: 10.3389/fphys.2022.822953 35185621 PMC8850775

[31] Huang J Zhang S Guo Y Zhai W. Difficulties and suggestions in the development of clinical practice guidelines for evidence-based acupuncture in knee osteoarthritis. J Tradit Chin Med. (2019) 60:1345–7. doi: 10.13288/j.11-2166/r.2019.15.019

[32] Li X (2021). Meta-analysis of acupotomy therapy for knee osteoarthritis. Hunan University of Traditional Chinese Medicine.

[33] Huang WJ Ren XX Xu ZH Xie W. Evaluation of application effect of hot ambao ironing in improving elderly patients with knee arthritis. J North Pharm. (2020) 17:129–31. doi: 10.3969/j.issn.1672-8351.2020.09.054

[34] Chen HJ Wang Y Jia XY Tang Q. Clinical observation of acupuncture combined with magnetic therapy in the treatment of knee osteoarthritis. J Clin Acupunct Moxibus. (2017) 33:26–8. doi: 10.3969/j.issn.1005-0779.2017.03.008

[35] Zhang J, Sun M. Acupuncture combined infrared irradiation treatment of knee osteoarthritis clinical observation. Contemp Med (2017) 23:7–9. doi: 10.3969/j.issn.1009-4393.2017.31.003

[36] Zou Z Zhu J Liao W. Systematic evaluation of therapeutic effect of aqua exercise therapy in elderly patients with knee osteoarthritis. Chin J Rehabil Med. (2011) 26:659–64. doi: 10.3969/j.issn.1001-1242.2011.07.014

[37] Li Y. Eff ect of Chinese medicine paraffi n in the treatment of “Gu-bi” patients with wind-cold and dampness syndromes and the change of the serum IL-37, IFN-γ, CD-62p, CD-41. J Changchun Univ Chin Med. (2018) 34:534–7. doi: 10.13463/j.cnki.cczyy.2018.03.039

[38] Yang Y. (2021). A meta-analysis of the efficacy of low intensity pulsed ultrasound in the treatment of knee osteoarthritis. Southern Medical University.

[39] Jones A Silva PG Silva AC Colucci M Tuffanin A Jardim JR. Impact of cane use on pain, function, general health and energy expenditure during gait in patients with knee osteoarthritis: a randomised controlled trial. Ann Rheum Dis. (2012) 71:172–9. doi: 10.1136/ard.2010.140178, PMID: 22128081

[40] Raja K Dewan N. Efficacy of knee braces and foot orthoses in conservative management of knee osteoarthritis: a systematic review. Am J Phys Med Rehabil. (2011) 90:247–62. doi: 10.1097/PHM.0b013e318206386b, PMID: 21273902

[41] Duivenvoorden T Brouwer RW van Raaij TM Verhagen AP Verhaar JAN Bierma-Zeinstra SMA. Braces and orthoses for treating osteoarthritis of the knee. Cochrane Database Syst Rev. (2015) 3:D4020. doi: 10.1002/14651858.CD004020.pub3 PMC7173742 25773267

[42] van Raaij TM Reijman M Brouwer RW Bierma-Zeinstra SMA Verhaar JAN. Medial knee osteoarthritis treated by insoles or braces: a randomized trial. Clin Orthop Relat Res. (2010) 468:1926–32. doi: 10.1007/s11999-010-1274-z, PMID: 20177839 PMC2881986

[43] Tong G. Clinical observation on“Haitongpi decoction”in treating severe keen osteoarthritis. Shanghai J Tradition Chin Med. (2012) 46:60–1.

[44] Li XC Shi XQ Xing RL Zhang NS Wang PM Xu B. Acupoint application treatment for knee osteoarthritis: a systematic review and meta analysis. J Nanjing Univ Tradition Chin Med. (2018) 34:421–5. doi: 10.14148/j.issn.1672-0482.2018.0421

[45] Ma X Yang G Yang C. Therapeutic effect of Chinese medicine iontophoresis on knee osteoarthritis: a meta-analysis. Chin Nurs Res. (2018) 32:3585–9. doi: 10.12102/j.issn.1009-6493.2018.22.025

[46] Xu JH Wang GD Xue RR Mo W Yu YQ Yin MC. A randomized control clinical study on Gutong plaster combined with exercise therapy in treatment for knee osteoarthritis. Chin J Med Guide. (2015) 12:1265–9.

[47] Zheng Y, Zhan H, Zhang H Niu SG Zhuang ZJ. Qi-zheng Qing-Peng slurry for treatment of the knee osteoarthritis: a random, controlled clinical research. Chin J Orthopaed Traumatol (2006) 19:316–7. doi: 10.3969/j.issn.1003-0034.2006.05.032

[48] He X Cao W Feng X. Clinical summary of 30 cases of knee osteoarthritis treated by Yunnan Baiyao tincture. Chin J Inform Tradition Chin Med. (2003) 10:45–6. doi: 10.3969/j.issn.1005-5304.2003.11.025

[49] Wolff Dylan G Christy C Brown Symone M. Topical nonsteroidal anti-inflammatory drugs in the treatment of knee osteoarthritis: a systematic review and meta-analysis. Phys Sportsmed. (2021) 49:381–91. doi: 10.1080/00913847.2021.1886573 33554694

[50] Rutjes AW Juni P Da CB da Costa BR Reichenbach S. Viscosupplementation for osteoarthritis of the knee: a systematic review and meta-analysis. Ann Intern Med. (2012) 157:180–91. doi: 10.7326/0003-4819-157-3-201208070-00473 22868835

[51] Vaishya R Pandit R Agarwal AK Vijay V. Intra-articular hyaluronic acid is superior to steroids in knee osteoarthritis: a comparative, randomized study. J Clin Orthop Trauma. (2017) 8:85–8. doi: 10.1016/j.jcot.2016.09.008, PMID: 28360505 PMC5359523

[52] Bannuru RR Natov NS Dasi UR Schmid CH McAlindon TE. Therapeutic trajectory following intra-articular hyaluronic acid injection in knee osteoarthritis--meta-analysis. Osteoarthr Cartil. (2011) 19:611–9. doi: 10.1016/j.joca.2010.09.014, PMID: 21443958 PMC11678314

[53] Han Y Huang H Pan J Lin J Zeng L Liang G. Meta-analysis comparing platelet-rich plasma vs hyaluronic acid injection in patients with knee osteoarthritis. Pain Med. (2019) 20:1418–29. doi: 10.1093/pm/pnz011 30849177 PMC6611633

[54] Zhao S Guo Z Yu Q. Evaluation of clinical efficacy and safety of dexamethasone palmitate injection in the treatment of knee osteoarthritis. J New Med. (2019) 50:115–22.

[55] Tang M Weng Z Shao L. Systematic review on the clinical curative effects and safety of traditional chinese medicine in the treatment of knee osteoarthritis. J Tradition Chin Orthoped Traumatol. (2014) 26:43–8.

[56] Pan JK Hong KH Liu J Xie H Huang HT Liang HD. Systematic review on the efficacy and safety of kidney-tonifying and blood-activating medicine for knee osteoarthritis. Chin J Tradition Chin Med Pharm. (2016) 31:5248–56.

[57] Xu X Liu W Xu S Li YK Zhang QW Huang HX. Clinical practice guidelines for knee osteoarthritis in integrated traditional chinese and western medicine. J Pract Med. (2021) 37:2827–33. doi: 10.3969/j.issn.1006

[58] Song M. (2021). Cluster analysis of traditional Chinese medicine syndromes and correlation study of clinical stages of knee osteoarthritis. China Academy of Chinese Medical Science.

[59] Huang L. (2021). Clinical study of Juanbi decoction in the treatment of knee osteoarthritis with cold and dampness impediment. Guangxi University of Chinese Medicine.

[60] Wang K Lou H Ye H. Simiao decoction combined with wrist and ankle acupuncture for the treatment of dampness-heat accumulation syndrome of mild to moderate knee osteoarthritis. J Tradition Chin Orthoped Traumatol. (2019) 31:41–3.

[61] Zhao W. (2015). Observation on curative effect of Taohong Siwu decoction on primary blood stasis type K0A [D]. Fujian University of Traditional Chinese Medicine.

[62] Wang K Zeng F Lu M Liao HZ. Meta analysis of the effect of Duhuo Jisheng decoction on knee osteoarthritis and inflammation of joint fluid. Clin J Tradition Chin Med. (2021) 33:883–9. doi: 10.16448/j.cjtcm.2021.0524

[63] Liu Z Fang R Meng Q. Effect of Bazhen decoction on TCM syndrome score and coagulation function of knee osteoarthritis patients after operation. Xinjiang J Tradition Chin Med. (2021) 39:1–3. doi: 10.3969/j.issn.1005-9202.2016.23.067

[64] Fu MR He LF Lyu J Xi JY Liu GY Xie YM. Meta-analysis of efficacy and safety of Hulisan capsules in treatment of knee osteoarthritis. Chin J Chin Mater Med. (2023) 47:1–12. doi: 10.19540/j.cnki.cjcmm.20220627.501 36472044

[65] Li J. Meta-analysis of efficacy and safety of Zhuanggujie capsule in the treatment of knee osteoarthritis. Shaanxi J Tradition Chin Med. (2022) 43:394–7. doi: 10.3969/J.ISSN.1000-7369.2022.03.030

[66] Zhao J Liang G Pan J Huang HT Yang WY Luo MH. Network Meta-analysis of oral Chinese patent medicine in treatment of knee osteoarthritis. Chin J Chin Mater Med. (2021) 46:981–99. doi: 10.19540/j.cnki.cjcmm.20200721.502 33645105

[67] Li J Cao X Song Y. A meta-analysis of treating knee osteoarthritis with the Xianling Gubao capsule. Clin J Chin Med. (2020) 12:143–8. doi: 10.3969/j.issn.1674-7860.2020.20.049

[68] Wang H Wu J Cai X Huang R Chen X Huang Q. Systematic evaluation and meta-analysis on the efficacy and safety of Biqi capsules in the treatment of knee osteoarthritis. Chin Med Herald. (2020) 17:127–31.

[69] Machado Gustavo C Maher Chris G Ferreira Paulo H Pinheiro MB Lin CW Day RO. Efficacy and safety of paracetamol for spinal pain and osteoarthritis: systematic review and meta-analysis of randomised placebo controlled trials. BMJ. (2015) 350:h1225. doi: 10.1136/bmj.h1225 25828856 PMC4381278

[70] Huang H Luo M Liang H Pan J Yang W Zeng L. Meta-analysis comparing celecoxib with diclofenac sodium in patients with knee osteoarthritis. Pain Med. (2021) 22:352–62. doi: 10.1093/pm/pnaa230, PMID: 32797224 PMC7901859

[71] Zhang X Li X Xiong Y Wang Y Wei J Zeng C. Efficacy and safety of tramadol for knee or hip osteoarthritis: a systematic review and network meta-analysis of randomized controlled trials. Arthritis Care Res. (2021) 75:158–65. doi: 10.1002/acr.24750 34251756

[72] Meng Z Liu J Zhou N. Efficacy and safety of the combination of glucosamine and chondroitin for knee osteoarthritis: a systematic review and meta-analysis. Arch Orthop Trauma Surg. (2022) 143:409–21. doi: 10.1007/s00402-021-04326-9, PMID: 35024906

[73] Singh JA Noorbaloochi S MacDonald R Maxwell LJ Cochrane Musculoskeletal Group. Chondroitin for osteoarthritis. Cochrane Database Syst Rev. (2015) 2016:CD005614. doi: 10.1002/14651858.CD005614.pub2 PMC4881293 25629804

[74] Kongtharvonskul J Anothaisintawee T McEvoy M Attia J Woratanarat P Thakkinstian A. Efficacy and safety of glucosamine, diacerein, and NSAIDs in osteoarthritis knee: a systematic review and network meta-analysis. Eur J Med Res. (2015) 20:24. doi: 10.1186/s40001-015-0115-7, PMID: 25889669 PMC4359794

[75] Thorlund JB Juhl CB Roos EM Lohmander LS. Arthroscopic surgery for degenerative knee: systematic review and meta-analysis of benefits and harms. BMJ. (2015) 350:h2747. doi: 10.1136/bmj.h2747, PMID: 26080045 PMC4469973

[76] Yu F Wang ZZ Yang B Wu J. A randomized trial of arthroscopic intervention for moderate and severe osteoarthritis of the knee. J Pract Orthopaed. (2014) 20:801–3.

[77] Singh N Trivedi V Kumar V Mishra NK Ahmad S Ayar SJ. A comparative study of osteoarthritis knee arthroscopy versus intra-articular platelet rich plasma injection: a randomised study. Malays Orthop J. (2022) 16:31–40. doi: 10.5704/MOJ.2207.004, PMID: 35992984 PMC9388795

[78] Wang B Yu N. Expert consensus on knee osteoarthritis ladder treatment (2018 edition). Expert Consensus Knee Osteoarth Ladder Treat. (2019) 13:124–30.

[79] Yu JA Liu XW Lian HY Liu KX Li ZT. Medial open-wedge tibial osteotomy versus lateral closed-wedge tibial osteotomy for unicompartmental knee osteoarthritis: a meta-analysis. Chin J Tissue Eng Res. (2023) 27:632–9. doi: 10.12307/2022.988

[80] Bei T Yang L Huang Q Wu J Liu J. Effectiveness of bone substitute materials in opening wedge high tibial osteotomy: a systematic review and meta-analysis. Ann Med. (2022) 54:565–77. doi: 10.1080/07853890.2022.2036805 35166617 PMC8856078

[81] Zhang L Wei M Liu A Shuwei G Zhiheng T Jianhao H. Comparison of HTO and UKA in the treatment of unicompartmental knee osteoarthritis: a meta analysis. Int J Biomed Eng. (2019) 2:143–9. doi: 10.3760/cma.j.issn.1673-4181.2019.02.010

[82] Li X (2019). A meta-analysis of high tibial osteotomy versus monocondylar replacement in the treatment of single-compartment knee osteoarthritis. Dalian Medical University.

[83] Huang Y Liu J Wang XS Xu HJ. Deep learning indications for high tibial osteotomy. Chin J Surg. (2020) 58:420–4. doi: 10.3760/cma.j.cn112139-20200228-00149 32498479

[84] Jing LZ Wang XL Liu K Wang XT Wang SS Yang JS. Clinical observation of arthroscopic clearance combined with distal femoral closure osteotomy in the treatment of knee valgus and osteoarthritis. Chin J Bone Joint Injury. (2020) 35:404–6.

[85] Du CY Wang YF Yang Y Wu LY Zhou X Wang YD. Biplane distal femoral osteotomy for valgus knee osteoarthritis. Orthoped J Chin. (2019) 27:1421–4.

[86] Zhang YQ Zhang Z Wu M Tao SL Yang YL Li Y. Evaluation of the efficacy of distal medial femoral closed osteotomy in the treatment of lateral interventricular osteoarthritis of the knee joint. Chin J Bone Joint Injury. (2019) 34:167–9. doi: 10.7531/j.issn.1672-9935.2019.02.019

[87] Liu A Cui Z Yu W. High tibial osteotomy versus unicompartmental knee arthroplasty for medial compartment osteoarthritis of the knee: a meta-analysis [J/OL]. Orthoped J Chin. (2022) 7:1–5. doi: 10.3977/j.issn.1005-8478.2022.07.10

[88] Liu AF Ma XL Cui ZS Yu WJ Guo TC. Unicompartmental knee arthroplasty versus total knee arthroplasty for knee osteoarthritis: a meta-analysis. Orthoped J Chin. (2021) 29:1955–60. doi: 10.3977/j.issn.1005-8478.2021.21.07

[89] Sheng D Song Q Bai XW Liang HS Deng Y Shu CK. Research progress of lateral unicondylar replacement of knee joint. Chin J Bone Joint Injury. (2022) 37:106–8. doi: 10.7531/j.issn.1672-9935.2022.01.037

[90] Sayah SM Karunaratne S Beckenkamp PR Horsley M Hancock MJ Hunter DJ. Clinical course of pain and function following total knee arthroplasty: a systematic review and meta-regression. J Arthroplast. (2021) 36:3993–4002.e37. doi: 10.1016/j.arth.2021.06.019, PMID: 34275710

[91] Shan L Shan B Suzuki A Nouh F Saxena A. Intermediate and long-term quality of life after total knee replacement: a systematic review and meta-analysis. J Bone Joint Surg Am. (2015) 97:156–68. doi: 10.2106/JBJS.M.00372, PMID: 25609443

[92] Skou ST Roos EM Laursen MB Rathleff MS Arendt-Nielsen L Simonsen O. A randomized, controlled trial of total knee replacement. N Engl J Med. (2015) 373:1597–606. doi: 10.1056/NEJMoa1505467 26488691

[93] Beswick AD Wylde V Gooberman-Hill R Blom A Dieppe P. What proportion of patients report long-term pain after total hip or knee replacement for osteoarthritis? A systematic review of prospective studies in unselected patients. BMJ Open. (2012) 2:e435. doi: 10.1136/bmjopen-2011-000435 PMC3289991 22357571

