# Supplementary material for: Guidelines for the diagnosis and treatment of knee osteoarthritis with integrative medicine based on traditional Chinese medicine
Source: Front Med (Lausanne). 2023 Oct 17;10:1260943. doi: 10.3389/fmed.2023.1260943 (PMC10617515; doi:10.3389/fmed.2023.1260943)
Supplement: Supplementary file 1 [file Data_Sheet_1.docx]

**METHODS**

The entire guideline development process is shown in Figure 1.

| 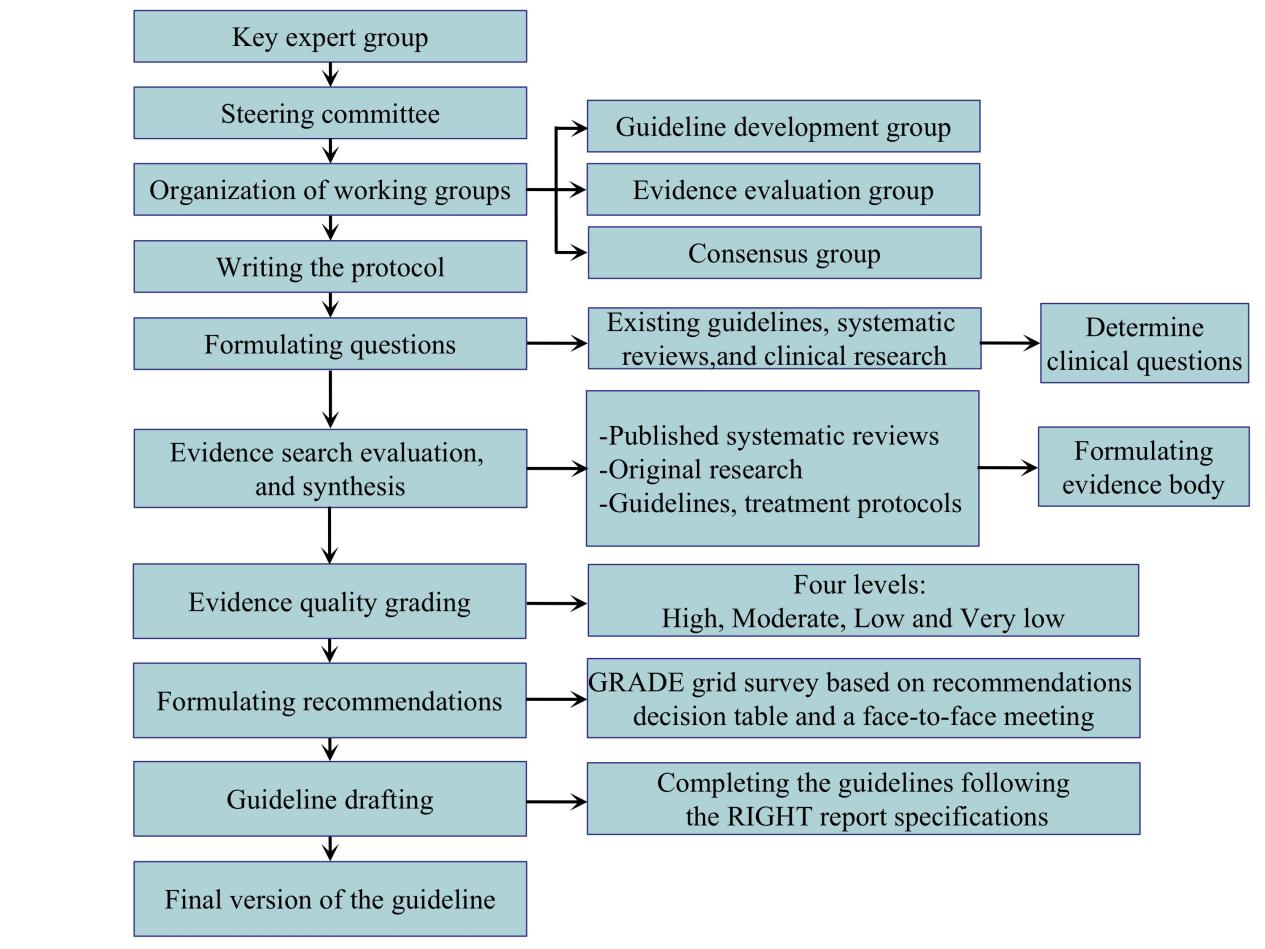 |
| --- |

Figure 1. Guideline development process.

**1. Constitution of the guideline development panel**

The guideline development panel comprised a chief expert group, a steering committee, a guideline development group, an evidence review group, and an expert consensus group. The consensus group was composed of multidisciplinary experts in traditional Chinese orthopedics, Western orthopedics, integrating Chinese and Western orthopedics, acupuncture and massage, sports medicine, traditional Chinese rehabilitation, clinical pharmacy, pharmacoeconomics, and nursing science.

**2. Declaration and management of conflict of interest**

Every member of the guidelines for the diagnosis and treatment of knee osteoarthritis with integrative medicine based on traditional Chinese medicine (TCM) working team was required to complete a declaration of conflicting interest form before participating in the guideline development. The steering committee reviewed all statements to determine whether a conflict of interest exists. For experts with potential financial or academic conflicts of interest, we managed them with WHO guidance. None of the panel members had anything to disclose. The guideline development group archives all conflicts of interest declaration forms and updates them according to the situation.

**3. Collection and determination of clinical questions**

Based on existing guidelines, systematic reviews, and clinical studies of knee osteoarthritis, the evidence review group systematically reviewed and collected clinical questions and outcome indicators. After deduplication, screening, and merging, we identified the specified main clinical questions and outcome indicators. After a face-to-face meeting, a consensus expert group determined important clinical questions and outcome indicators through an online questionnaire. The rules were as follows: (1) score 1−7 points for the clinical questions initially included, 1 point meaning very unimportant, 7 points meaning very important; and (2) the outcome indicators initially included are scored from 1 to 9 points. A total of 7−9 points indicated critical, 4−6 points indicated important, and 1−3 points indicated generally. According to the survey results and the steering committee’s discussion, certain clinical issues were selected, including three back ground questions and six prospect questions. The three background questions included diagnostic criteria and clinical stages of knee osteoarthritis, TCM syndrome differentiation, and classification of knee osteoarthritis. More details about prospect questions can be found in the main text, and can also be further contacted with the key author’s E-mail lingfengzeng@gzucm.edu.cn. Finally, we determined patient-important outcomes based on the scores.

**4. Evidence collection**

Systematic reviews, meta-analyses, and randomized controlled trials (RCTs) were included. The evidence review group followed the principles of high-quality systematic review production, deconstructed the clinical questions according to the PICOS (population, intervention, comparison, outcome, and study design) principle, and formulated the systematic review based on the clinical questions in this guideline.

Systematic searches were conducted using topic terms and free words. We searched the following databases for systematic reviews and meta-analyses, network meta-analyses, and RCTs: MEDLINE (via PubMed), Embase, the Cochrane Library, the China National Knowledge Infrastructure (CNKI), WanFang, VIP, and the China Biology Medicine disc (CBM). The search period was from the establishment of the database to June 30th, 2022, and only English and Chinese were accepted as languages of publication.

Inclusion criteria: (1) Patients: confirmed knee osteoarthritis cases (treatment) or high-risk population (prevention); (2) Intervention: TCM or integrative medicine; (3) Comparison: Western medicine treatment generally consisted of NSAIDs, patient education and others was determined based on the latest guidelines for the diagnosis and treatment of knee osteoarthritis; (4) Outcomes: patient-important outcomes included the VAS scores for pain, Knee range of motion, Knee osteoarthritis WOMAC Scale (pain, stiffness, improvement in joint function), et al. Supplementary outcomes were also considered in the included RCTs. The patient-important outcomes were determined based on expert consensus; (5) study design: RCTs.

Exclusion criteria: (1) TCM prescriptions with unclear ingredients; (2) multiple TCM prescriptions in which the combined effect was indistinguishable from a single therapy effect; (3) protocols, etc.

**5. Evidence evaluation and grading**

In systematic reviews and meta-analyses (network meta-analyses), RCTs were evaluated using the AMSTAR scale and the Cochrane Collaboration tool for assessing the risk of bias (ROB) scale, respectively.

We used the GRADE system to evaluate evidence quality and recommendations. Two researchers independently extracted data and assessed the quality of the studies. The related documents are included: 1) Quality assessment of the included studies; 2) the GRADE evidence profile, which contains detailed information about the quality of evidence assessment and effect sizes of each clinical evidence.

**6. Formulation of recommendations**

Based on the evidence included, the guideline development group initially developed appropriate recommendations for clinical practice, taking into full account the balance of benefits and harms, the certainty of the evidence, costs, clinical feasibility, accessibility, and clinical acceptability. Then, the GRADE grid method was used to reach a consensus for all recommendations. The consensus rule was that if the votes for any box other than “no clear recommendation” exceeded 50%, a consensus was reached, and the direction and strength of recommendation was deemed directly determined; if the total votes for two squares on one side of “no clear recommendation” exceeded 70%, the result was also regarded as a consensus, the recommendation direction could be determined, and the recommendation strength was directly defined as “weak” (sometime guideline panel may use terms such as “conditional” instead of weak). Those recommendations for which a consensus was reached could be modified by the consensus group as well. After completing the GRADE grid survey, a face-to-face meeting was held on July 10th, 2022, with the specified recommendations reached by consensus. Following the specifications in the RIGHT report, the guideline development group completed the guidelines. Then, the draft guideline was sent to the experts (representing 26 institutions in China) who did not participate in the guideline development process for suggestions. Finally, we further improved the draft based on modification suggestions.

**DISSEMINATION AND PROMOTION OF GUIDELINES**

Following publication, the guideline will be disseminated and promoted as follows: (1) introduce and interpret the guideline in relevant academic conferences; (2) organize orthopedic surgeons, rehabilitation physicians, nursing staff and other related medical workers to learn the relevant contents of the guideline; (3) distribute to public media outlets such as WeChat.

**GUIDELINE UPDATE PLAN**

We plan to monitor clinical research evidence regarding the role of TCM. It is up to the steering committee to evaluate and determine whether to initiate or update guideline recommendations when there is new research evidence that could change clinical practice or previous recommendations.
