# Supplementary material for: Guidelines for the diagnosis and treatment of knee osteoarthritis with integrative medicine based on traditional Chinese medicine
Source: Front Med (Lausanne). 2023 Oct 17;10:1260943. doi: 10.3389/fmed.2023.1260943 (PMC10617515; doi:10.3389/fmed.2023.1260943)
Supplement: Supplementary file 2 [file Data_Sheet_2.docx]

**Annex A**

**(Normative)**

**Grading of evidence in the scheme**

**(Refer to the Status and Strategies of evidence-based guidelines for clinical practice in traditional Chinese medicine)**

**Table A.1 Classification of literature basis**

| **Grading** | **Criteria** |
| --- | --- |
| Grade Ⅰ | Large samples, randomized studies, clear results, and low false positive or false negative errors |
| Grade Ⅱ | Small samples, randomized studies, inconclusive results, high false positive and/or false negative errors |
| Grade Ⅲ | Non-randomized, contemporaneous controlled trials and expert consensus based on ancient literature |
| Grade Ⅳ | Non-random, historically controlled and contemporary expert consensus |
| Grade Ⅴ | Case reports, uncontrolled studies and expert opinion |

Note: ① In the "research topic classification" of the standard, large sample and small sample are defined as: Large sample: ≥100 high-quality single randomized controlled trial reports or systematic review reports. Small sample: <100 cases of high quality single paper. "Expert consensus based on ancient literature" in level III refers to those recorded in ancient medical books, used in successive generations, and reached consensus on contemporary expert opinions. In level IV, "contemporary expert consensus" refers to a consensus of contemporary expert survey opinions. "Expert opinion" in Level V refers only to individual expert opinion.

**Table A.2 AMSTAR Scale (Systematic Review and Meta-analysis)**

| **Item** | **Description and Explanation** |
| --- | --- |
| 1 | Are pre-design options available? Research questions and inclusion exclusion criteria should be identified prior to the systematic review |
| 2 | Are the selection and data extraction of included studies reproducible? There should be at least two independent data extractors, and a process of agreeing on reasonable differences of opinion should be used. |
| 3 | Are extensive and comprehensive literature searches implemented? Search at least 2 electronic databases. Search reports must include the year as well as databases such as Central, EMbase, and MEDLINE. The keyword/subject term used must be stated and a search strategy should be provided if possible. Up-to-date catalogues, reviews, textbooks, professional registries, or specialists in a particular field should be consulted for additional searches, as well as for post-bibliographic references. |
| 4 | Has publication status been taken into account in the inclusion criteria, such as grey literature? It should be stated that the reviewer's search is not limited by the publication type; It should be stated whether the evaluator excluded documents based on their publication, such as language. |
| 5 | Is a list of included and excluded studies available? A list of included and excluded studies should be provided. |
| 6 | Are the characteristics of the included studies described? The data extracted from the original study should include information on subjects, interventions and outcome measures, and be summarized in forms such as tabulation; A range of characteristics included in the study should be reported, such as age, race, sex, relevant socioeconomic data, disease status, disease duration, and severity. |
| 7 | Is the science of the included study evaluated and reported? Pre-designed evaluation methods should be provided, such as in therapeutic studies, and whether evaluators use randomization, double-blind, placebo-controlled, and assigned concealment as evaluation criteria should be accounted for in the relevant criteria for other types of studies. |
| 8 | Was the science of the included study properly applied to the derivation of the conclusions? The rigor and science of the methodology should be considered in the analysis of the results and the derivation of the conclusions. It also needs to be clear when forming recommendations. |
| 9 | Were the methods used to synthesize the inclusion findings appropriate? For synthesis results, statistical tests should be used to determine whether the included studies are compostable and to assess their heterogeneity (e.g. Chi-squared test). If heterogeneity exists, random-effects models should be used, and/or the clinical suitability of the resultant results should be considered, such as whether the resultant results are sensitive. |
| 10 | Was the possibility of publication bias assessed? Publication bias assessment should be supplemented by a graph, such as a funnel plot, as well as other feasible detection methods and/or statistical tests, such as Egger regression. |
| 11 | Is the conflict of interest stated? Potential funding sources for systematic review and inclusion in the study should be clearly accounted for? |

Each item evaluation result can be divided into "yes", "no", "unclear" or "not mentioned", and given a score, such as "yes" is 1 point, "no", "unclear" or "not mentioned" is 0 points, a total of 11 points, AMSTAR scale score 0-4 is classified as low quality, 5-8 is classified as medium quality, 9-11 is classified as high quality. More than 5 points of literature were selected as evidence.

**Table A.3 Cochrane Bias risk Assessment tool guidelines**

| **Bias type** | | **Bias risk assessment level** | | |
| --- | --- | --- | --- | --- |
|  |  | **Low risk bias** | **High risk bias** | **unclerar** |
| **Selection bias** | **The generation of random sequences** | Researchers have descriptions of random components in the process of generating random sequences. For example, using a table of random numbers; Using computer random number generators; Toss a coin; A sealed card or envelope; Casting son; Draw lots. | Odd even number or date of birth; Date of admission (or day of week), etc., or direct non-random classification of subjects, such as grouping according to the following factors: doctor's judgment; The patient's performance, etc. | There is not enough information to determine the above two levels. |
|  | **Allocation hiding** | Central randomization (including randomization based on telephone, network, pharmacy control), etc. | The distribution envelope is not properly secured (e.g. not sealed, transparent, not a random sequence); Alternate or cycle, etc. | There is not enough information to determine the above two levels. |
| **Implementation bias** | **Researchers and subjects were blinded** | No or incomplete blinding, but the review authors determined that the outcome was unlikely to be blinded by images lacking blinding; Blind the subjects, the principal researchers. | The outcome may be blind missing images; The subjects and the researchers in charge of recruitment are blinded, but it is possible to break the blindness, and the outcome may be blind images. | Without sufficient information to determine the above two levels; Not mentioned. |
| **Measurement bias** | **The study outcome was evaluated by blind method** | The outcome was evaluated blind, but the review authors determined that the outcome was unlikely to be blinded by the missing images; Blind evaluation of the outcome is guaranteed, and it is unlikely to be broken blind. | The review authors determined that the outcome might be blinded by the lack of images; Blind evaluation of outcomes is performed, but the blindness may have been broken, and the outcome measurement may be subject to blind missing images. | Without sufficient information to determine the above two levels; Not mentioned. |
| **Follow-up bias** | **Integrity of the resulting data** | There were no missing data in the outcome. The reason of missing outcome index is unlikely to be related to the truth value of outcome; The missing outcome measures were balanced across groups for similar reasons. For binary outcome measures, the proportion of missing outcome measures with the risk of observed events was not sufficient to determine that their estimate of the intervention effect had clinically relevant images; For the sequential outcome measure, the effect size of the missing outcome was insufficient to determine that it had clinically relevant images for the observed effect size. The missing data were filled in with appropriate methods. | The reason of missing outcome indicators may be related to the truth value of outcome, and the number or reason of missing indicators is inconsistent between groups. For binary outcome measures, the proportion of missing outcome measures and the risk of observed events were sufficient to determine that their estimates of the intervention effect had clinically relevant images; For the sequential outcome measure, the effect size of the missing outcome was sufficient to introduce clinically relevant bias into the observed effects; When a large number of interventions violated random assignment, the "as treatment" strategy was applied to the analysis; The missing data was filled in by an inappropriate method. | The information for follow-up or exclusion in the report is insufficient to determine the above two levels; Not mentioned. |
| **Reporting bias** | | Research protocols are available and all pre-stated outcomes of concern are reported; Research protocols are not available, but published reports contain all expected results, including those stated in advance. | Not all pre-stated major outcomes have been reported; One or more of the major primary outcome measures used undeclared measures, methods, or subdatasets. One or more primary outcome measures are not stated in advance; One or more of the main outcome measures of concern to the review investigators were not reported completely and could not be included in the meta-analysis. The study did not report the desired primary outcome. | There is not enough information to determine the above two levels. |
| **other** | | No other bias was apparent. | There is a potential bias associated with a particular study design; There is fraud; Other questions. | Insufficient information to assess whether there is a significant risk of bias; There is no good reason or evidence that the existing question would introduce bias. |

**Table A.4 Modified Jadad Scale (RCT)**

| **Items** |  | **Points and content** |
| --- | --- | --- |
| **The generation of random sequences** | 1 Appropriate  2 Not clear  3 Inappropriate | 2 points: computer-generated random numbers or similar methods  1 point: Randomised trial but no description of the method of randomisation  0 points: If the method of alternating allocation is used, such as single and even signs |
| **Randomized hiding** | 1 Appropriate  2 Not clear  3 Inappropriate | 2 points: Center or pharmacy control of the allocation protocol, or use of sequence-numbered containers, on-site computer control, sealed opaque envelopes, or other methods that make the allocation sequence unknown to clinicians and subjects  1 point: Indicates only the use of a random number table or other random allocation scheme  0 points: alternating assignments, case numbers, number of Sundays, open random number tables, serially coded envelopes, and any measures that do not prevent the predictability of grouping |
| **Blind method** | 1 Appropriate  2 Not clear  3 Inappropriate | 2 points: Completely consistent placebo tablets or similar methods were used  1 point: The trial was presented blinded but the method was not described  0 points: Failure to use double-blind or blind methods is inappropriate, such as tablet and injection comparison |
| **Withdrawal and loss of follow-up** | 1 Yes  2 None | 1 point: Describes the number and reasons for withdrawal and loss to follow-up  0 points: The number or reason for withdrawal and loss to follow-up is not described |

Literature with a score greater than or equal to 3 on the modified Jadad scale was selected for evidence.

**Table A.5 MINORS entry scores (non-randomized controlled trials)**

| **Items** | **Prompt** |
| --- | --- |
| 1. The purpose of the study is clearly given | The issues defined should be precise and relevant to the available literature |
| 2. Inclusion of patient coherence | All potentially prospective patients (meeting the inclusion criteria) were included during the study (no exclusion or reasons for exclusion given) |
| 3. Collection of expected data | Data set in the study protocol developed prior to the start of the study were collected |
| 4. The endpoint indicators can appropriately reflect the purpose of the study | Clear explanations of the criteria used to evaluate outcomes consistent with the defined questions, and endpoints should be assessed on the basis of intention-to-treat analysis |
| 5. Objectivity of endpoint index evaluation | Evaluators were single-blinded for objective endpoint indicators and double-blinded for subjective endpoints. Otherwise, reasons for not blinding the assessment should be given |
| 6. Whether the follow-up time is adequate | Follow-up should be long enough. This enabled the assessment of endpoint indicators and possible adverse events |
| 7. Loss to follow-up rate is less than 5% | All patients should be followed. Otherwise, the proportion of patients lost to follow-up cannot exceed the proportion of patients reflecting the primary endpoint indicator |
| 8. Whether the sample size is estimated | The incidence of events according to the expected outcome. Sample sizes with 95% confidence intervals for detecting different outcomes were calculated: information was provided to enable comparison of expected and actual results in terms of statistically significant differences and estimated power levels |
| Clauses 9-12 were used to evaluate additional criteria for studies with control groups |  |
| 9. Whether the selection of the control group is appropriate | For diagnostic tests, it should be the "gold standard" for diagnosis; For therapeutic intervention trials, it should be the most residential intervention available from published studies |
| 10. Whether the control group is synchronized | The control group and the experimental group should be conducted at the same time (non-historical control) |
| 11. Whether baselines between groups are comparable | Unlike the study endpoints, the baseline criteria for starting points for the control and experimental groups should be similar. There were no confounding factors that could bias the interpretation of results |
| 12. Whether the statistical analysis is appropriate | Whether the statistics used to calculate confidence intervals or risk ratios (RR) match the type of study |

There are 12 evaluation indicators, each of which is divided into 0-2 points. The first 8 studies were for no control group, with a maximum score of 16 points; The last 4 studies were for studies with a control group, with the highest score of 24 points. A score of 0 indicates that it is not reported; A score of 1 indicates that it was reported but not sufficiently informed; A score of 2 indicates that it was reported and sufficiently informative. Literature with an overall score of 13 or greater was selected as evidence of therapeutic advice. Many of the titles of the literature are randomized controlled, but the content is non-randomized in nature, such as grouping in the order of visits, etc., which should be classified as non-randomized trials. If there are obvious quality problems, such as discrepancies between the number of cases in the categorical statistical sample and the total sample number of cases in the group, and treatment reports with poor theoretical analysis, they should be directly discharged without being scored.

**Table A.6 NOS Evaluation Criteria (Cohort Study)**

| **Column** | **Items** | **Criteria** | **Score** |
| --- | --- | --- | --- |
| **Study population selection** | Representativeness of the exposure group (1 point) | ①True representation of the characteristics of the exposure group in the population*;  ②To a certain extent represents the characteristics of the exposure group in the population*;  ③Select a certain type of population, such as nurses and volunteers;④The source of the exposure group is not described |  |
|  | Representativeness of the non-exposed group (1 point) | ①From the same population as the exposure group*;  ②From different populations with the exposure group;  ③Non-exposing group sources were not described |  |
|  | Methods for determining exposure factors (1 point) | ①Fixed archival records (e.g. surgical records)*;  ②Use structured interviews*;  ③Reports written by the research subjects themselves;  ④Not described |  |
|  | Identification of outcomes that were not to be observed at the start of the study (1 point) | ①Yes*；②No |  |
| **Comparisons between groups** | Design and statistical analysis considering the comparability of exposed and non-exposed groups (2 points) | ①The study controlled for the most important confounding factors*; The study controlled for any other confounding factors* |  |
| **Result measurement** | Whether the study evaluated the results adequately (1 point) | ①Blinded oppositional evaluation*; ②Have archival records*;  ③Self-reporting;  ④Not described |  |
|  | Whether follow-up after results was long enough (1 point) | ①Yes (appropriate follow-up time prescribed prior to evaluation)*;  ②No |  |
|  | Adequacy of follow-up in the exposure group and non-violent groups (1 point) | ①Complete follow-up*;  ②A small number of participants were lost to follow-up without introducing bias (specifying loss to follow-up or describing loss to follow-up)*;  ③There is loss to follow-up (prescribed loss rate) but not described;  ④Loss to follow-up was not described |  |
| Note: * is the scoring point; The highest score of 2 points for intergroup comparability is awarded | | | |

**Table A.7 Classification of evidence for programmes**

| **Evidence level** | **Evaluation criteria** |
| --- | --- |
| **Level I** | It is supported by at least 2 grade I studies |
| **Level II** | It was supported by only one grade I study |
| **Level III** | Only the results of the grade II study are supported |
| **Level IV** | It is supported by at least one grade III study |
| **Level V** | Only the results of the study at grade IV or V are supported |

**Appendix B**

**(Normative)**

**A description of the recommended strength**

**Table B Description of recommended strengths**

| **Recommended strength** | **Evaluation indicators** |
| --- | --- |
| **Highly recommended** | For clinicians, most clinicians choose to use this recommendation; For patients, the vast majority of patients will take the recommendation, and only a few will not; For policymakers, recommendations are adopted as policy in most cases. |
| **Weak recommendation** | Clinicians should recognize that different patients have their own protocols and need to help each patient make decisions that reflect their values and preferences; For patients, most patients will adopt recommendations, but many patients will not use them; For policymakers, policy development requires substantive discussion and the involvement of a wide range of stakeholders. |
